# Revealing arginine-cysteine and glycine-cysteine NOS linkages by a systematic re-evaluation of protein structures

**DOI:** 10.1038/s42004-025-01535-w

**Published:** 2025-05-13

**Authors:** Sophia Bazzi, Sharareh Sayyad

**Affiliations:** 1 Institute of Physical Chemistry, Georg-August University Göttingen, Tammannstraße 6, Göttingen, D-37077 Germany; 2 Department of Mathematics and Statistics, Washington State University, Pullman, WA 99164-3113 USA; 3 Mathematical Institute, Georg-August University Göttingen, Bunsenstraße 3-5, Göttingen, 37073 Germany

**Keywords:** X-ray crystallography, Cheminformatics, Proteins, Enzymes

## Abstract

Nitrogen-oxygen-sulfur (NOS) linkages act as allosteric redox switches, modulating enzymatic activity in response to redox fluctuations. While NOS linkages in proteins were once assumed to occur only between lysine and cysteine, our investigation shows that these bonds extend beyond the well–studied lysine-NOS-cysteine examples. By systematically analyzing over 86,000 high–resolution X-ray protein structures, we uncovered 69 additional NOS bonds, including arginine-NOS-cysteine and glycine-NOS-cysteine. Our pipeline integrates machine learning, quantum–mechanical calculations, and high-resolution X-ray crystallographic data to systematically detect these subtle covalent interactions and identify key predictive descriptors for their formation. The discovery of these previously unrecognized linkages broadens the scope of protein chemistry and may enable targeted modulation in drug design and protein engineering. Although our study focuses on NOS linkages, the flexibility of this methodology allows for the investigation of a wide range of chemical bonds and covalent modifications, including structurally resolvable posttranslational modifications (PTMs). By revisiting and re-examining well-established protein models, this work underscores how systematic data-driven approaches can uncover hidden aspects of protein chemistry and inspire deeper insights into protein function and stability.

## Abstract (web summary)

Nitrogen-oxygen-sulfur (NOS) linkages serve as crucial allosteric redox switches in proteins, yet their diversity beyond lysine-cysteine pairs remains underexplored. Here, the authors employ machine learning and quantum-mechanical calculations to identify 69 NOS bonds, expanding protein chemistry’s landscape and offering new avenues for drug design and protein engineering.

## Introduction

In 2021, efforts to develop treatments for gonorrhea brought renewed attention to an overlooked covalent linkage^1,2^. This Lys-NOS-Cys linkage, characterized as a redox switch, features a nitrogen-oxygen-sulfur bond bridging the side-chain nitrogen of lysine and the sulfur atom of cysteine^2^. Initially identified within the transaldolase enzyme of Neisseria gonorrhoeae, this structural motif has since emerged across diverse organisms^3^. These findings are particularly significant because Lys-NOS-Cys linkages help regulate protein structure and function in response to shifts in the cellular redox environment^3^. Our continued identification of chemical bonds in protein structures suggests additional overlooked NOS linkages or other chemical interactions in protein data banks, highlighting the need for a closer examination. Since no dedicated tools currently exist for this type of discovery, developing methods for the identification of (new types of) NOS linkages could spark a wider effort to explore novel chemical interactions in proteins, shedding light on uncharted aspects of protein chemistry.

A major challenge in this field is the absence of systematic chemical bond discovery algorithms, compounded by experimental constraints for obtaining structural details of amino acid residues and their interactions. For example, X-ray crystallography is a powerful method for characterizing protein structures; however, the outcome of this approach depends heavily on the accurate translation of electron density maps into atomic models. This procedure relies on assumptions of chemical feasibility and geometric constraints derived from known protein chemistry. Although these assumptions generally hold, deviations can result in modeling errors or the omission of previously unrecognized chemical bonds. Aside from a single study^1^, Lys-NOS-Cys linkages have frequently been misclassified as “close contacts” in validation reports or entirely overlooked in protein structures. Some of these bonds were misidentified as Lys-CH_2_-Cys linkages^4^. Furthermore, older protein data bank entries may lack detailed structural information due to limitations in experimental measurements, making it inevitable to revisit and re-evaluate the fine structure of their chemical bonds. Reassessing these structures with automated algorithms and machine learning (ML) techniques could improve the classification of chemical bonds and reveal previously unrecognized interactions within protein data banks.

Accurate modeling of chemical bonds and interactions within protein structures is a fundamental prerequisite for expanding the possibilities of drug discovery and protein engineering. This enables directed modifications and precise tuning of protein conformation, stability, and function to achieve desired biological or therapeutic objectives. This gives a rationale for the re-examination of protein structures that have already been determined so that one may possibly identify chemical bonds and interactions that have been overlooked. Some recently discovered bonds may stem from radiation damage or specific crystallization conditions. Understanding these possibilities is crucial for recognizing new chemical features in protein structures, understanding environment-induced reactivity, and improving protein refinement libraries. Integrating these insights deepens our understanding of protein chemistry and enhances the reliability of structural data, providing a robust platform for future innovation in biomedical research.

Alongside conventional biochemical approaches, ML and artificial intelligence techniques are gaining momentum in addressing complex problems in biochemistry^5^. The rapidly evolving field of scientific ML enables the analysis of high-dimensional data and accelerates the discovery of underlying structures across diverse biochemical systems^6–14^. Among the expanding range of computational methods for exploring biological data, pattern recognition, and unsupervised learning methods are particularly appealing for their affordable computational costs and reasonable efficiency. These ML-driven techniques have proven valuable in deciphering protein function and dynamics^15–20^ and in identifying novel protein interactions^21–25^, offering deeper insights into the high-dimensional structure of biological data.

These studies collectively demonstrate that ML techniques are powerful tools for uncovering previously unknown structures and interactions, offering new insights into complex biological systems. Despite the critical need to characterize chemical bonds and identify potentially hidden interactions within protein data banks, no systematic approach currently exists to accomplish these tasks effectively. To address this gap, we introduce a methodology to characterize and categorize NOS bonds. Our objectives are (i) to extend the discovery of NOS bonds beyond lysine and cysteine by incorporating interactions between cysteine and a select set of other residues, (ii) to streamline the bond-finding process through automation and ML techniques, and (iii) to establish a generic roadmap that accelerates future bond discoveries. Our algorithm identifies *sixty-five* new candidates of Lys-NOS-Cys, *two* Gly-NOS-Cys, and *two* Arg-NOS-Cys linkages. This is one of the primary outcomes of the present work. In addition, we introduce a set of robust descriptors for each of these chemical bonds, marking a key methodological advance in our study. These descriptors not only permit precise identification and characterization of NOS linkages but also serve as a valuable foundation for future studies exploring analogous chemical interactions.

## Results and discussion

**Fig. 1:**
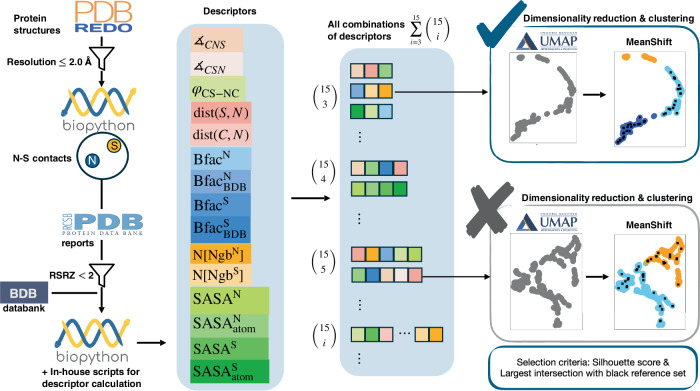
Workflow of data acquisition and ML methods in *SimplifiedBondfinder* algorithm. The *SimplifiedBondfinder* algorithm collects data from three different protein data banks, namely, PDB^36^, PDB-REDO^35^, and BDB^30^. The collected data undergo various constraints to filter out reliable datasets. For each sample in the dataset, fifteen different descriptors are gathered. The descriptors are a collection of angles (∡), torsion angles (*φ*), interatomic distances (dist), atomic B-factor values from the protein data bank (Bfac) and BDB (Bfac_BDB_), numerized (*N*) neighbor amino acids to the target residues (Ngb^N/S^), and solvent accessible surface areas of the target atoms (SASA_atom_) and their corresponding residues (SASA^N/S^). All possible descriptor sets of size three or greater are generated from this list. For each set, Uniform Manifold Approximation and Projection (UMAP) dimensionality reduction^28^ is applied, followed by mean-shift clustering. The best minimal set of descriptors is finally selected when their Silhouette score exceeds 0.5 and an insignificant intersection of reference points, reported in ref. ^3^, with one of the clusters is observed.

In this work, we showcase how our numerical approach unveils new, highly probable candidates for NOS (nitrogen-oxygen-sulfur) linkages. Before diving into these findings, we first outline the conceptual basis of our method, dubbed “*SimplifiedBondfinder*”, which is visually summarized in Fig. 1.

Protein structures are often highly complex, with various geometrical, structural, and additional parameters, such as solvent-accessible surface area (SASA) and B-factors (Bfac). These parameters that offer insights into specific biological and chemical properties are archived in protein data banks. The first step in our algorithm collects this information while enforcing criteria related to resolution, atom types, and interatomic distances. This results in a curated dataset residing on a high-dimensional manifold, ready for subsequent processing. For each entry in this dataset, a collection of *fifteen* descriptors, such as (torsion) angles and distances, is gathered, as detailed in Fig. 1. We also incorporate a set of experimentally verified NOS linkages^3^ into our dataset. We hypothesize that any putative new NOS bonds should occupy the same high-dimensional manifold as the known Lys-NOS-Cys linkages (reference points). These verified linkages show strong electron density peaks between sulfur and nitrogen atoms and can be refined with an NOS linkage. Note that the dimensionality of our manifold (≥3) is determined by the chosen descriptors from a pool of all possible combinations of the fifteen descriptors summed up to $$\mathop{\sum }\nolimits_{i = 3}^{15}\left(\begin{array}{l}15\\ i\end{array}\right)$$ descriptor sets.

Additionally, for each residue pair, we retrieve “Too-close contacts” from the full validation reports on the RCSB PDB website^26^. These contacts flag non-bonded atom pairs falling below a specified distance threshold, indicating potential steric clashes of van der Waals shells as described in the validation report user guide^27^. Notably, ~45% of the Lys-NOS-Cys linkages previously reported in ref. ^2^ are flagged as “close contacts” in their corresponding validation reports (see Supplementary Data 1). This observation suggests that such “Too-close contacts” may occasionally reflect missing covalent linkages rather than steric clashes of van der Waals shells, particularly for sulfur-nitrogen pairs, and could thus be redefined to accommodate cases involving an overlooked bond. Therefore, revising the definition of “Too-close contacts” could be beneficial. This is because these interactions do not strictly involve non-bonded atom pairs but rather pairs that are modeled as non-bonded.

A critical step in our workflow involves applying a dimensionality reduction method that optimally preserves the intrinsic topological and geometric properties of the high-dimensional data manifold. UMAP ^28^ accomplishes this by preserving local neighborhood relationships while approximating global connectivity through a fuzzy topological representation. This approach ensures that essential structural features are retained in the lower-dimensional embedding, facilitating meaningful downstream analysis. To make our approach generalizable, it is also important that the manifold learning technique does not impose strict numerical restrictions on the embedding dimensions, allowing for diverse protein properties to be incorporated. Furthermore, UMAP distance metrics have demonstrated superior performance compared to other dimensionality reduction techniques, such as PCA, random orthonormal projections, and t-SNE^24^ in the identification of known protein complexes, pathways, and novel protein-protein interactions.

To identify the key descriptors underlying NOS linkage formation, we first project the dataset into a low-dimensional space (2-3 dimensions) using UMAP, followed by mean-shift clustering. Notably, alternative algorithms (e.g., k-means^29^) yield comparable outcomes. This workflow systematically evaluates descriptor combinations while isolating well-separated clusters containing the highest number of reference points. We define the minimal embedding dimension that supports such robust clustering as the set of “critical descriptors” for identifying NOS linkages.

When discussing these critical descriptors, it is important to note that geometric and contextual features surrounding any given chemical bond can vary substantially in different protein structures. Influencing factors range from local protein environment and residue identity to sequence distance (for inter-residue bonds; see Supplementary Fig. 4 in ref. ^3^), as well as experimental conditions like resolution and X-ray dose in crystallographic data.

These characteristics can also differ within a single protein structure, which shows the dynamic and complex nature of chemical bonds in proteins. Despite this variability, it is essential to identify common patterns and shared features for chemical bonds with the same atomic composition. These shared features will not only enable the discovery of novel protein bonds but also provide a basis for predicting their likelihood and stability. Such predictive insights can be a major contributing factor for the refinement of entries in protein data banks, guiding protein engineering efforts, and improving computational modeling.

### Descriptor selection with ML techniques

**Fig. 2:**
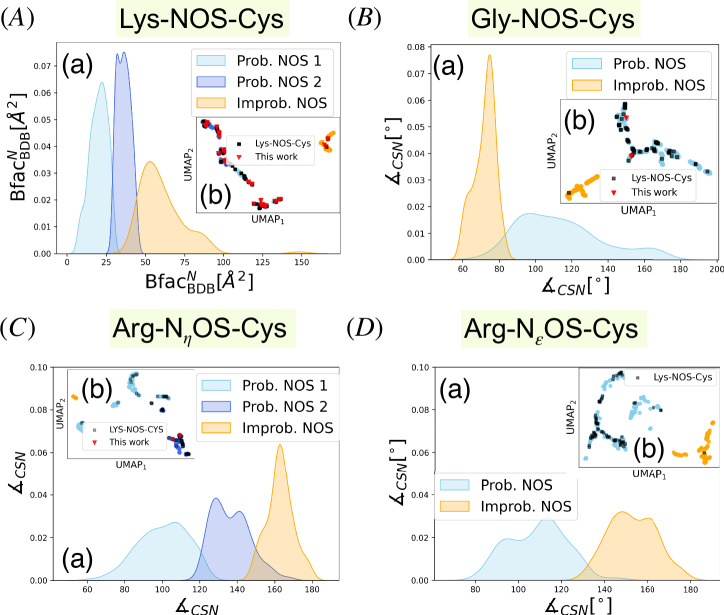
Density distribution plots and UMAP results obtained from the *SimplifiedBondfinder* algorithm. Density distributions and UMAP plots for covalent linkages of Lys-NOS-Cys (**A**), Gly-NOS-Cys (**B**), Arg-N_*η*_OS-Cys (**C**), and Arg-N_*ε*_OS-Cys (**D**). Probable NOS-bond candidates are depicted in sky blue and royal blue, while improbable candidates are shown in orange. The black square points denote the reference dataset presented in ref. ^3^. The red inverted triangles depict our newly found sets of proteins exhibiting NOS linkages. For the Lys-NOS-Cys dataset, each cluster in (**A**)(b) consists of *n* = 271 (sky blue), *n* = 155 (royal blue), and *n* = 101 (orange) samples. The Silhouette score in (**A**)(b) is 0.61. The mode of ${{\rm{Bfac}}}_{{\rm{BDB}}}^{{\rm{N}}}$ for probable samples is 22.21 (sky blue), 36.24 (royal blue), and 52.84 (orange) in (**A**)(a). For Gly-NOS-Cys in (**B**), the cluster sizes are *n* = 271 (sky blue), and *n* =  81 (orange). The Silhouette score is 0.58. Dominant modes of density distributions in (**B**)(a) for ${\measuredangle}_{{\rm{CSN}}}$ are 96.63, and 74.68 for sky blue and orange samples, respectively. For Arg-N_*η*_OS-Cys in (**C**), the sizes of generated clusters are *n* = 266 (sky blue), *n* = 147 (royal blue), and *n* = 54 (orange). The associated Silhouette score is 0.55. The dominant modes of density distributions in (**C**)(a) for ${\measuredangle}_{{\rm{CSN}}}$ are 106.79, 128.61, and 162.92 for sky blue, royal blue, and orange samples, respectively. For Arg-N_*ε*_OS-Cys dataset in (**D**), the size of clusters are *n* = 235 (sky blue) and *n* = 83 (orange). The associated Silhouette score is 0.56. The dominant modes of density distributions for ${\measuredangle}_{{\rm{CSN}}}$ in (**D**)(a) are 113.38 and 148.07 for probable and improbable samples, respectively.

As illustrated in Fig. 1 and outlined above, our algorithm performs UMAP dimensionality reduction followed by mean-shift clustering in three- to fifteen-dimensional descriptor spaces. We restricted our analysis to residues that have well-defined B-factors in the PDB files with consistent B-factors (BDB)^30^. The procedure then selects the minimal descriptor set that yields distinct clusters, at least one of which intersects with all or most of the experimentally verified NOS linkages (reference points)^3^. Clusters containing these reference points are termed “probable clusters” (shown in sky blue or royal blue in Fig. 2). In contrast, ones containing none or very few reference points are termed “improbable clusters,” depicted in orange. The reference points themselves are indicated by black squares in the figures.

To demonstrate the effectiveness of this approach, we first apply it to data that potentially host Lys-NOS-Cys linkages. This dataset, comprising 527 lysine-cysteine pairs, also includes experimentally verified NOS linkages. The automated procedure identifies a minimal descriptor set composed of B-factors for the nitrogen atom (${{\rm{Bfac}}}_{{\rm{BDB}}}^{{\rm{N}}}$)^30^, together with the numbers of neighboring residues within a 4 Å radius of the *C*_*α*_ atoms of lysine (Ngb^N^) and cysteine (Ngb^S^); see Supplementary Fig. S1Aa–f. As shown in Fig. 2Ab, UMAP dimensionality reduction followed by mean-shift clustering results in three distinct clusters. Two of these clusters (total size 426) collectively host the majority of reference points, thus forming the probable clusters, whereas the third improbable cluster (size 101) contains only two of the 74 reference linkages, indicating a lower probability of exhibiting NOS linkages.

Our overarching objective is to determine whether NOS bonds can form beyond well-established Lys-Cys pairs, specifically exploring the cysteine sulfur atom in combination with both backbone and side-chain nitrogen atoms. While a comprehensive survey of all sulfur-nitrogen interactions lies outside the scope of this work, we focus on the most statistically abundant pairings in these two subsets (see Supplementary Tables S1–II). Notably, among backbone nitrogen contacts, Gly-Cys ranks second only to Cys-Cys, which generally forms disulfide bonds. This makes glycine our primary candidate. Meanwhile, among side-chain nitrogen contacts, Arg-Cys is the most frequently observed. For certain atoms lacking an associated B-factor in the BDB, we exclude residue pairs containing them from the dataset. In future studies, we intend to broaden our analysis to additional residue types listed in the Supplementary Tables that may harbor overlooked NOS linkages.

**Fig. 3:**
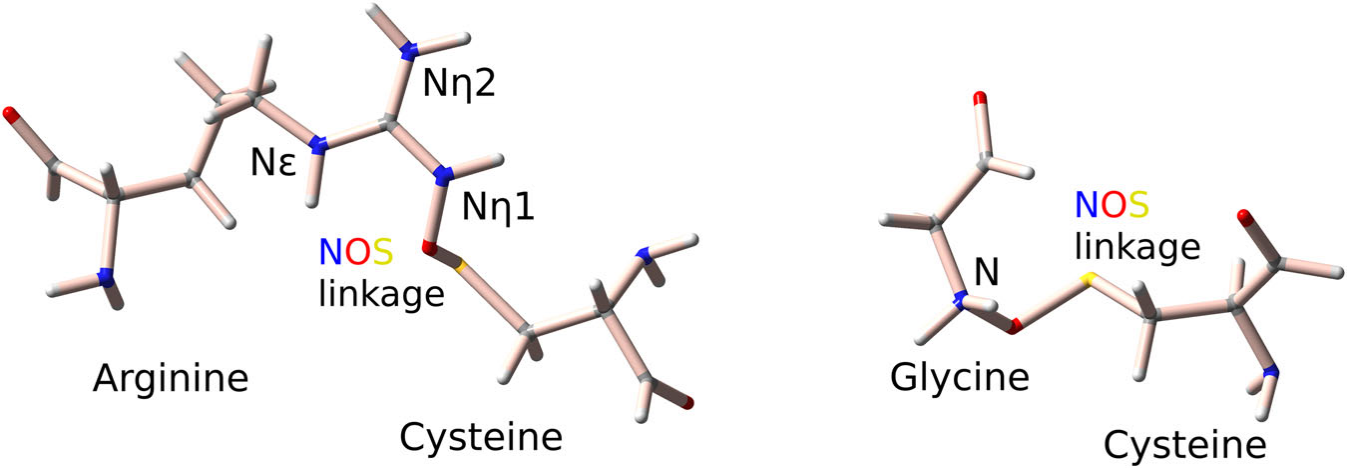
Schematic representation of NOS linkages between arginine-cysteine and glycine-cysteine. Selected protonation states of the residues are shown, with atom names involved in the nitrogen-oxygen-sulfur (NOS) linkage labeled. For details on Lys-NOS-Cys linkages, refer to ref. ^34^.

We extend our analysis to a dataset of 313 glycine-cysteine pairs to explore potential Gly-NOS-Cys linkages. A schematic representation of these linkages is shown in Fig. 3. Here, the minimal descriptor set comprises the B-factors for the sulfur-bearing residue (${{\rm{Bfac}}}_{{\rm{BDB}}}^{S}$), the sulfur-nitrogen distance (dist (*S*, *N*)), and the carbon-sulfur-nitrogen angle (${\measuredangle}_{{\rm{CSN}}}$); see Supplementary Fig. S1Ba–f. As shown in Fig. 2Bb, mean-shift clustering partitions the data into a probable cluster of 271 entries (containing 73 of the 74 reference points) and an improbable cluster of 81 entries (with only one reference point), indicating a clear demarcation between likely and unlikely Gly-NOS-Cys linkages.

We further apply our method to identify the key descriptors for predicting the formation of NOS linkages between arginine and cysteine residues (Arg-NOS-Cys). As shown in Fig. 3, the arginine side chain has two types of nitrogen atoms, namely, N_*η*_ and N_*ε*_, which differ in terms of the geometrical features and chemical properties. Thus, we analyze the datasets for N_*η*_ (Arg-N_*η*_OS-Cys) and for N_*ε*_ (Arg-N_*ε*_-Cys) independently.

For Arg-N_*η*_OS-Cys, the selected descriptors include the solvent-accessible surface area (SASA) of the nitrogen-bearing residue (SASA^N^), the ${\measuredangle}_{{\rm{CSN}}}$, and the neighbor residues to sulfur(Ngb^S^) and to nitrogen (Ngb^N^), as shown in Supplementary Fig. S1Ca–j. UMAP dimensionality reduction and mean-shift clustering yield three clusters as shown in Fig. 2Cb, two of which with a combined size of 413 contain 73 of the 74 reference points and are therefore designated probable; the third, improbable cluster, with a size of 54, contains just one reference point.

Applying the same pipeline to a dataset of 240 Arg-N_*ε*_OS-Cys pairs identifies a descriptor set involving ${{\rm{Bfac}}}_{{\rm{BDB}}}^{{\rm{S}}}$, SASA^S^, SASA of nitrogen atom (${{\rm{SASA}}}_{{\rm{atom}}}^{{\rm{N}}}$), ${\measuredangle}_{{\rm{CSN}}}$ and ∡_CNS_ as shown in Supplementary Fig. S1Da–o. This combination yields two clusters containing 235 (probable) and 83 (improbable) samples, respectively, see Fig. 2Db.

### Biochemical significance of the multidimensional descriptor space

Here, we discuss the biochemical relevance of the key descriptors that permit the separation of NOS and non-NOS linkages within the minimal descriptor sets identified by our algorithm. Figure 2Aa displays the density distribution of ${{\rm{Bfac}}}_{{\rm{BDB}}}^{{\rm{N}}}$ for each cluster. The mode of each distribution is clearly distinct, at 20.76 Å^2^, and 35.97 Å^2^ for the probable clusters and 57.85 Å^2^ for the improbable cluster, indicating that a large number of probable samples (bluish) have ${{\rm{Bfac}}}_{{\rm{BDB}}}^{{\rm{N}}}$ values below 44.5 Å^2^.

This suggests greater rigidity in these segments compared to improbable samples, as B-factors generally correlate with the flexibility of atoms or regions within protein structures^31^. Furthermore, since structural integrity and stability are crucial for the proper function of residues in active sites^31^, amino acid residues in these regions typically exhibit lower B-factors, indicating their role in enzymatic activity. Lys-NOS-Cys linkages are frequently found in key structural motifs, such as Schiff bases and metal-binding sites, where stable interactions are required for maintaining structural integrity^2,3^. We emphasize that while lower B-factors of the involved atoms can be an indication of NOS bonding, they could also reflect involvement in other nitrogen-sulfur interactions.

The modes of ${{\rm{Bfac}}}_{{\rm{BDB}}}^{{\rm{S}}}$ (see Supplementary Fig. S2) exhibit similar trends as ${{\rm{Bfac}}}_{{\rm{BDB}}}^{{\rm{N}}}$ with modes observed at 24.04 Å^2^ and, 34.09 Å^2^ for the probable clusters and 42.35 Å^2^ for the improbable cluster. However, there is a significant overlap in the overall distributions between the clusters.

It is worth noting that we also included B-factors directly obtained from the protein data bank when building our dataset. However, our algorithm suggests that the values of B-factors from the BDB better discern probable samples hosting Lys-NOS-Cys linkages from improbable ones; see Supplementary Fig. S2. With respect to this observation, which is consistent throughout our whole study, we emphasize that B-factors from BDB could facilitate future bond classification.

Although ${{\rm{Bfac}}}_{{\rm{BDB}}}^{{\rm{N}}}$ is the primary contributing factor in distinguishing the two clusters, the correlation between ${{\rm{Bfac}}}_{{\rm{BDB}}}^{{\rm{N}}}$ and neighboring residues in {Ngb^S^, Ngb^N^} reveals a clear separation between the bluish and orange data points, as illustrated in Supplementary Fig. S1Aa–f. Consequently, the combination of these three descriptors constitutes the minimal descriptor space necessary for identifying additional Lys-NOS-Cys candidates.

The dataset exhibiting probable Gly-NOS-Cys linkages is characterized by a distinct set of effective descriptors, with ${\measuredangle}_{{\rm{CSN}}}$ emerging as the primary descriptor for distinguishing probable clusters of NOS linkages. As shown in Fig. 2Ba the majority of probable samples show ${\measuredangle}_{{\rm{CSN}}} > 8{0}^{\circ }$. Notably, optimized Gly-NOS-Cys complexes demonstrate an ${\measuredangle}_{{\rm{CSN}}}$ value around 94° (see Table 1) in agreement with the primary mode of the probable cluster (96. 6°). The correlation between ${\measuredangle}_{{\rm{CSN}}}$ and other elements of the descriptor space, i.e., ${{\rm{Bfac}}}_{{\rm{BDB}}}^{{\rm{S}}}$ and dist(*S*, *N*) is essential for forming well-separated accumulations of sample points for each cluster, as shown in Supplementary Fig. S1Ba–f.

**Table 1:** Selected structural parameters and root-mean-square deviations (RMSDs) for optimized complexes containing NOS linkage

Complex	PDB	dist(S,N) [Å]	∡_CSN_ [°]	∡_CNS_ [°]	∡_NOS_ [°]	RMSD [Å]
Gly-NOS-Cys	6PGD	2.70	94.02	95.11	118.93	0.2421
	6T3X	2.69	94.67	93.89	118.00	0.3610
Gly-NOS-Cys^+1^	6PGD	2.67	95.87	96.29	116.09	0.2445
	6T3X	2.65	95.46	95.61	115.54	0.3239
ARG-N_*η*_OS-Cys (N_*η*2_H_2_, N_*η*1_)	3MWB	2.61	122.59	121.26	122.66	0.5669
	3G2K	2.62	91.20	130.18	113.65	0.2773
ARG-N_*η*_OS-Cys^+1^ (N_*η*2_H_2_, N_*η*1_H_1_)	3MWB	2.62	110.87	109.32	113.20	0.6178
	3G2K	2.61	90.80	134.76	114.14	0.2313
ARG-N_*η*_OS-Cys (N_*η*2_H, N_*η*1_H)	3MWB	2.64	106.33	104.08	114.71	0.6522
	3G2K	2.61	91.19	136.33	113.96	0.1948

RMSD values represent the deviation between the optimized geometry and the final refined NOS-containing structure. In our optimization protocol, backbone atoms were constrained to their positions in the NOS-refined structure, except for the glycine backbone nitrogen, which is involved in the NOS linkage. This approach ensures that observed differences primarily reflect local side-chain rearrangements in both RMSD calculations.

For Gly-NOS-Cys linkages, the distributions of ${{\rm{Bfac}}}_{{\rm{BDB}}}^{{\rm{S}}}$ shown in Supplementary Fig. S1Ba, indicates that unlike the Lys-NOS-Cys samples, the majority of probable and improbable clusters exhibit similar values of ${{\rm{Bfac}}}_{{\rm{BDB}}}^{{\rm{S}}}$. These values are largely consistent with ${{\rm{Bfac}}}_{{\rm{BDB}}}^{{\rm{N}}}$ as presented in Supplementary Fig. S3. However, for the majority of cases in both probable and improbable clusters, the values of ${{\rm{Bfac}}}_{{\rm{BDB}}}^{{\rm{S}}}$ and ${{\rm{Bfac}}}_{{\rm{BDB}}}^{{\rm{N}}}$ are lower than 50 Å^2^. Generally speaking, the observed intermediate values of ${{\rm{Bfac}}}_{{\rm{BDB}}}^{{\rm{N}}}$ can be attributed to the backbone rigidity of glycine^31,32^, as the nitrogen in this dataset is a backbone nitrogen. The intermediate values of ${{\rm{Bfac}}}_{{\rm{BDB}}}^{{\rm{S}}}$, which are comparable to the probable clusters of lysine and cysteine samples, could have several reasons. For instance, in 44% of all samples, the sulfur atom of cysteine is involved in metal coordination, disulfide bonds, and other types of covalent bonding (see Supplementary Table III). In the remaining structures, it forms a ring-shaped configuration with the nitrogen atom of glycine. This unique arrangement likely contributes to increased sulfur-atom rigidity and warrants additional experimental investigation.

Having examined the dataset involving sulfur and nitrogen atoms in glycine, we now turn our attention to the second group of samples that may host Arg-N_*η*_OS-Cys linkages. The pair plot of the four-dimensional (descriptor) space in the case of ARG-N_*η*_OS-CYS is presented in Supplementary Fig. S1Ca–j, resulting in three distinct clusters. Specifically, as highlighted in Fig. 2Ca the modes of ${\measuredangle}_{{\rm{CSN}}}$ for the probable clusters are located at 104.01° (sky blue) and 133.28° (royal blue), closely matching the calculated values of optimized Arg-NOS-Cys clusters (104.08°–136.33°) as indicated in Table 1. In contrast, for the improbable cluster, the mode of ${\measuredangle}_{{\rm{CSN}}}$ at 178.27° (orange), deviates significantly from the expected quantum mechanical values (Table 1). This suggests that these structures are unlikely to accommodate NOS linkages and further highlights the advantage of ${\measuredangle}_{{\rm{CSN}}}$ as a key descriptor for distinguishing probable from improbable Arg-N_*η*_OS-Cys interactions. These findings emphasize the importance of geometric parameters in the identification and validation of biologically relevant NOS linkages.

For Arg-N_*ε*_OS-Cys, the ${\measuredangle}_{{\rm{CSN}}}$ remains a key determinant in distinguishing likely vs. unlikely NOS linkages, as shown in Fig. 2Da. Specifically, for ${\measuredangle}_{{\rm{CSN}}}$, we observe two modes of distributions at 95.67° (sky blue) and 115.26° for the probable cluster. The former mode aligns perfectly with the calculated values (95.96°, 99.47°, 99.79°; see Supplementary Table 1), while the latter is slightly larger than the calculated values. In contrast, the mode of distribution for ∡_CNS_ in the Supplementary Fig. S1Do is located at 148.90°, deviating by approximately 50° from the calculated angles. The overall minimal descriptor set for Arg-N_*ε*_OS-Cys consists of five descriptors: ${{\rm{Bfac}}}_{{\rm{BDB}}}^{{\rm{S}}}$, SASA^S^, ${{\rm{SASA}}}_{{\rm{atom}}}^{{\rm{N}}}$, ${\measuredangle}_{{\rm{CSN}}}$, and ∡_CNS_. The density plots for these descriptors are shown in Supplementary Fig. S1D.

### Clusters analysis

**Fig. 4:**
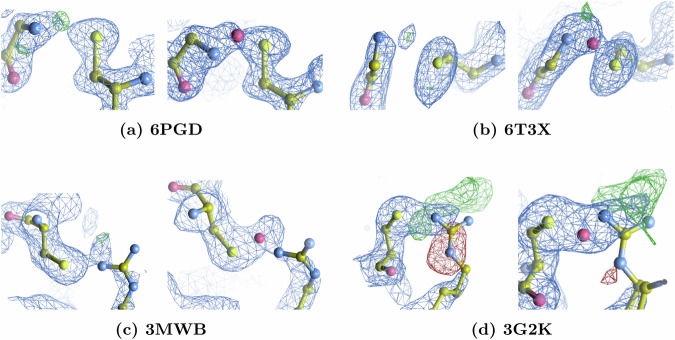
Comparison of electron density maps before and after refinement for the predicted NOS bridges. In each paired panel, the left image shows the pre--refinement state obtained after 10 cycles of simulated annealing, while the right image displays the final refined model. The corresponding 2mFo-DFc electron density maps are shown in blue at a contour level of 1 *σ*. The mFo-DFc difference electron density maps are shown in green at a contour level of 3.5 *σ*. **a** Suggests an NOS linkage between the terminal residues of Gly30 and Cys334 in a protein complex involving a truncated WD repeat-containing protein 5 (WDR5) (PDB: 6PGD). **b** Suggests an NOS linkage between Gly-5, as part of the prepended N-terminal extension (GSHMAS), and Cys35 of a truncated human cytomegalovirus pUL50-pUL53 complex (PDB: 6T3X). **c** Suggests an NOS linkage between Cys34 and Arg43 of Prephenate dehydratase in complex with L-Phe from Arthrobacter aurescens (PDB: 3MWB). **d** Suggests an NOS linkage between Cys783 and Arg786 of 1-(beta-D-glucopyranosyl)-4-substituted-1,2,3-triazole (PDB: 3G2K). The structural parameters for all panels from X-ray crystallographic models are presented in Supplementary Table VIII. Refinement led to substantial improvements in *R*_work_, *R*_free_, and clashscore, and effectively eliminated spurious positive difference density. Detailed validation statistics are provided in Supplementary Tables IX–XII.

Having identified the probable clusters, we then manually verified all entries within both the probable and improbable datasets using Coot^33^, focusing on the presence or absence of positive electron density between sulfur and nitrogen atoms at 3*σ*. We also compared the coordinates of any unexplained positive electron density with the quantum-mechanical estimates in Table 1 to assess the likelihood of an intervening oxygen atom consistent with an NOS linkage. Minor discrepancies from the optimized structural parameters can arise because structures in PDB-REDO and other databases are refined without accommodating NOS linkages. The positive peaks in Fig. 4 indicate regions of increased electron density that may correspond to an intervening oxygen atom between sulfur and nitrogen. None of the residue pairs in the improbable clusters showed evidence of an NOS bond in their electron density maps. Notably, only four of the 65 newly identified Lys-NOS-Cys linkages fell into the improbable clusters, confirming the efficacy of our classification. Finally, no significant positive difference density was observed to indicate Arg-N_*ε*_OS-Cys linkages.

From this assessment we detected *sixty-five* Lys-NOS-Cys (see Supplementary Table V), *two* Gly-NOS-Cys (Fig. 4(a) and (b)), and *two* Arg-N_*η*_OS-Cys linkages (Fig. 4(c) and (d)). These newly identified Gly-NOS-Cys and *Arg-NOS-Cys* linkages are marked by red inverted triangles in Fig. 2 (B)(b), and Fig. 2 (C)(b) and were further confirmed through explicit modeling and re-refinement. Incorporating NOS linkages not only accounted for previously unexplained positive electron density (Fig. 4) but also improved *R*_work_, *R*_free_, and clashscores, indicating better agreement between the experimental data and the refined models (see Supplementary Tables IX-XII). In the case of 3G2K (see Fig. 4(d)), the original structure showed a negative electron density peak surrounding the arginine side chain, which was substantially diminished after refinement by assigning an alternative conformation to arginine. 3G2K also shows a positive difference peak near the arginine side chain in both models. Given the peak’s large magnitude and the presence of DMSO, it may represent an unmodeled solvent molecule not accounted for in the current model. We did not re-refine the recently discovered Lys-NOS-Cys linkages because their existence is already established in the literature^2,3^.

## Structural and thermodynamic validation

To further confirm the Arg-NOS-Cys and Gly-NOS-Cys linkages proposed by our analysis, we combined quantum-mechanical geometry optimizations with thermodynamic assessments on four representative protein complexes (6PGD, 6T3X, 3MWB, and 3G2K). These systems were examined in multiple protonation states to account for the chemical variability likely present in vivo, though specific protonation schemes were chosen primarily for completeness rather than direct biological relevance.

### Structural validation

Each protein was optimized under two distinct conditions: one permitting the formation of the bridging oxygen (NOS) and another excluding it to simulate potential noncovalent interactions. In both scenarios, all backbone atoms were fixed to either the coordinates of the NOS-refined structure (for NOS complexes) or the corresponding PDB-REDO coordinates (for non-NOS complexes). The only exception was the glycine backbone nitrogen, which was allowed to relax due to its direct involvement in the NOS bond. Table 1 and Supplementary Table VI summarize key structural parameters for both NOS and non-NOS geometries, including the sulfur-nitrogen distance dist(*S*, *N*), the angles ∡*CSN* and ∡*CNS*, and RMSD values relative to the reference structure.

In NOS-linked optimized models, the S-N distance ranged from 2.61 to 2.70 Å, closely matching the 2.63-2.89 Å interval found in the original PDB-REDO structures. By contrast, removing the bridging oxygen caused a significant increase in S-N separation (3.36–4.26 Å), indicating that the shorter S-N distance observed experimentally is consistent with an intervening oxygen atom. The angle ∡*CSN*, previously identified by our ML pipeline as a critical descriptor, also remained near experimental values in NOS complexes but varied widely in non-NOS optimizations. These differences are reflected in lower RMSD values (0.19-0.65 Å) for the NOS-linked complexes compared to non-NOS complexes (0.27-0.91 Å), underscoring that incorporation of an NOS bond aligns more closely with the crystal structure than noncovalent alternatives.

Moreover, introducing quantum-mechanically optimized NOS parameters into our refinement protocols improved the fit to electron density maps, reducing unexplained peaks and confirming the plausibility of these linkages. Given the high sensitivity of refinement outcomes to subtle variations in bond lengths and angles, this consistency provides the most compelling evidence that these contacts represent authentic covalent NOS linkages rather than noncovalent associations.

### Thermodynamic assessment

To further evaluate these linkages, we computed Gibbs free energies (Δ*G*) under varying protonation states (Supplementary Table VII). Although we do not present a complete mechanistic framework, these comparisons clarify whether substituting a hydrogen with an oxygen to form an NOS bond is thermodynamically favorable. The detailed multi-step mechanism reported for Lys-NOS-Cys^34^ underscores the complexity of such reactions in biological systems, and a similar in-depth analysis is required to fully capture the formation pathway for Arg-NOS-Cys and Gly-NOS-Cys under physiological conditions. All computed Δ*G* values are negative, indicating that substituting one hydrogen by oxygen to form an NOS bond is thermodynamically feasible in the modeled states. However, the magnitude of Δ*G* varied considerably with the protonation state and between arginine- and glycine–derived complexes. In both systems, a neutral Gly or Arg is preferred over a positively charged state. Glycine-based complexes exhibited somewhat higher Δ*G* values. While these values still imply a thermodynamically favorable linkage, they are systematically less exergonic than the corresponding arginine complexes.

Taken together, these structural results provide convergent evidence that Arg-NOS-Cys and Gly-NOS-Cys bonds are plausible covalent linkages rather than simple nonbonded contacts. The consistency between quantum-mechanically optimized geometries and refined crystallographic data, along with negative free energies of formation, strongly indicates that these linkages are both structurally and energetically viable in relevant protein contexts.

## Conclusion

Our systematic analysis of over 86,000 high-resolution protein structures reveals that nitrogen-oxygen-sulfur (NOS) linkages exhibit greater diversity than previously recognized. Beyond the well-characterized lysine-cysteine pairs, we identify new types of NOS linkages involving arginine-cysteine and glycine-cysteine. Re-refining representative protein structures with an intervening oxygen atom between sulfur and nitrogen in these newly identified NOS linkages improved crystallographic metrics, supporting their existence.

Our methodology rests on two key components. First, we constructed targeted datasets focusing on specific chemical bonds, applying stringent criteria such as atom types, interatomic distances, and structure resolution. Second, we used ML techniques to explore these high-dimensional datasets, identify effective structural descriptors, and predict potential sites of covalent linkage formation. By systematically searching for common patterns and shared features, we uncovered 69 NOS linkages and the corresponding predictive descriptors.

Beyond the direct implications for protein chemistry, our findings highlight the importance of reexamining structural datasets using modern computational approaches. By developing a data-driven pipeline, we establish a robust framework for detecting and characterizing key chemical interactions such as disulfide bonds, backbone modifications, noncanonical metal coordination, and ligand-binding sites. In future work, we plan to expand NOS linkage analyses to encompass all residue combinations, extend this methodology to other posttranslational modifications (PTMs), and adapt these techniques for a wider range of experimental techniques beyond X-ray crystallography. We anticipate that this approach will drive the discovery of new biochemical motifs, offering insights with potential applications in protein engineering, drug design, and synthetic biology.

## Method

In the following section, we present our automated algorithm and our approach for manually inspecting suggested NOS linkages.

### *SimplifiedBondfinder* algorithm

We present details on our *SimplifiedBondfinder* algorithm, enabling us to categorize and identify prospective undetected chemical bonds in protein structures; see also the workflow of this algorithm in Fig. 1. In the following, we provide further details on data acquisition, descriptor optimization and clustering analysis within our algorithm.

#### Dataset acquisition

We start with collecting all structures from the PDB-REDO (as of Jan. 2024)^35^. The PDB-REDO updates static structures in the PDB to align with contemporary crystallographic standards by re-refining and optimizing them, thereby enhancing their accuracy and reliability compared to their original PDB entries^36^. From the initial database of *170,251* proteins, we initiate the first step of our automated dataset generation using multiple interconnected functions. Biopython (v 1.79) was utilized for structure parsing (using MMCIFParser and PDBParse) and computing additional atomic and residue properties^37,38^. Only X-ray-determined structures (*170,127 proteins*) were parsed. Given our focus on fine chemical bond details, we retained only protein structures with resolution values of less than or equal to 2 Å for improved prediction accuracy, resulting in *86,491* structures for further analysis.

To investigate specific chemical bonds, we established criteria based on constituent atom types, residue names, inter-atomic distances, and occupation numbers. For NOS linkages involving sulfur (S) and nitrogen (N) atoms in standard residues, we constrain the inter-atomic distance of S-N (dist(*S*, *N*)) to be less than or equal to 3.2 Å corresponding to the cutoff for the covalent interactions between lysine and cysteine^3^. We impose an occupation number threshold to be >0.8 to exclude atoms with high positional uncertainty. Application of these criteria identified *25,462* N-S contacts.

To ensure that the quality of depicted target atoms is satisfactory, we apply a real-space-R-value Z-score (RSRZ) threshold to be <2.0, which identifies reliable fits to the data in real space^39^. This criterion reduced our dataset to *23,129* N-S contacts. This dataset encompasses a diverse range of amino acid pairs, with cysteine and methionine as the sulfur-containing residues. A comprehensive breakdown of residue pairs and their frequencies appears in Supplementary Tables I-II. As detailed in these tables, our investigation focuses primarily on two types of interactions involving cysteine: (1) between the sulfur atom of cysteine and the backbone nitrogen of glycine, and (2) between the sulfur atom of cysteine and the side chain nitrogens of arginine and lysine. These pairs were selected based on their statistical abundance in the 23,129 N-S contacts, while close contacts that are likely formed for reasons other than NOS bond formation were excluded. For example, in cysteine-cysteine contacts involving backbone nitrogens, the proximity is often due to disulfide bridges rather than NOS formation. In the case of histidine-cysteine contacts, histidine is frequently involved in metal coordination, which can lead to unusual close contacts with other residues and produce ambiguous electron density that is difficult to interpret as an NOS bond. Although it is, in principle, possible for NOS bonds to form these alternative contacts, in the present study, we focused exclusively on the most unambiguous and statistically abundant cases to provide a robust proof-of-concept. This deliberate focus establishes a firm foundation for our approach, while future work will extend the search to include additional residue types and contacts, as detailed in Supplementary Tables I-II.

Our code acquires information on RSRZ after downloading the “full validation report”^40^ accessible from the RCSB PDB website^26^. The next step of our algorithm involves extracting structural parameters, including angles (∡_*CSN*_, ∡_CNS_), torsion angle (*φ*_CS-NC_), and other distances (dist(C, N), dist(S, N)) using the NeighborSearch module in Biopython. The carbon atom here refers to the closest carbon covalently bound to the nitrogen and sulfur atoms.

Using Bio.PDB.SASA, our code further calculates values of the Solvent Accessible Surface Area (SASA) for target atoms and the corresponding residues^41^. To have a parameter for the mobility of target atoms in our analysis, we also include atomic B-factors (Bfac). These values are gathered from two data banks, namely, the RCSB PDB^42^ and a data bank of PDB files with consistent B-factors (BDB)^30^. For certain atoms, the associated B-factor is absent in the BDB. If one of these atoms is present in the residue pairs, we exclude that pair from the dataset.

Up to this stage, we have gathered *fifteen* distinct descriptors for each prospective chemical bond in protein structures. It is challenging to analyze this high-dimensional dataset using classical approaches. To overcome this difficulty, we employ dimensionality reduction techniques. This allows us to find highly probable positive candidates and identify the smallest set of descriptors that distinguishes these candidates from the rest. Our employed ML algorithm combines an unsupervised Uniform Manifold Approximation, and Projection (UMAP)^28^ with a clustering (mean-shift) method. Through this ML approach, our algorithm introduces clusters that host probable and improbable candidates for our target chemical bonds.

It is important to emphasize that our ML framework is fundamentally unsupervised. While UMAP is employed here in an unsupervised manner, the approach could be extended to a semi-supervised setting by integrating experimentally verified labels into the training process. Such an extension would be particularly advantageous when a substantial number of known chemical bonds are available, enabling the algorithm to utilize prior knowledge for enhanced classification accuracy. However, this study focuses on the discovery of novel bonding patterns, such as NOS linkages, where labeled data are either sparse or entirely absent. As a result, we confine our analysis to the unsupervised approach and do not explore semi-supervised outcomes in this work.

#### Descriptor optimization and clustering analysis

Accomplishing the goal of finding an effective set of descriptors is a challenging task. To automate this process, we apply UMAP with a maximum of three embedding dimensions, followed by mean-shift clustering on $$\mathop{\sum }\nolimits_{i = 3}^{15}\left(\begin{array}{l}15\\ i\end{array}\right)=32642$$ combinations of all possible sets of descriptors. We then obtain a Silhouette Score^43^ for all three-dimensional embedding coordinates to evaluate the clustering quality of each combination. The algorithm outputs the clusters, the Silhouette Score, and the reference target linkages in each cluster. Each candidate is identified by the names of target atoms, corresponding residue names, residue numbers, chains, and PDB ID to distinguish between all target atoms within a protein (see Supplementary Code 1). To find the final and the smallest feature space, we utilized several criteria, including the value of the Silhouette Score, the number of clusters produced by each feature space, and the distribution of reference target linkages within these clusters. Specifically, we aimed to identify a feature space that effectively segmented the data into two/three distinctive clusters with a Silhouette Score ≥0.5, ideally containing none of the reference target linkages in one cluster, referred to as the improbable cluster. In practice, a minimal number of reference samples in this cluster is acceptable. The remaining clusters containing all or most reference target linkages are named probable clusters. This systematic approach enables us to identify feature spaces optimized for distinguishing between pairs of our target atoms likely to form new chemical bonds and those less likely to host such bonds (see Supplementary Code 1, Supplementary Code 2). Once we determine a small set of descriptors that reliably separate these cases, incorporating additional descriptors becomes unnecessary. A minimal set offers practical advantages in terms of computational efficiency and interpretability. This enhances the predictive accuracy of our approach in identifying novel chemical bond formations within protein structures.

### Rationale for UMAP embedding dimensions

The choice of embedding dimension in UMAP varies depending on the dataset and the geometric/topological properties of its original high-dimensional manifold. Ideally, dimensionality reduction should retain the essential structural features of the original manifold while minimizing information loss. In practice, two- or three-dimensional embeddings are the most interpretable, as they allow for straightforward visualization and assessment of clustering quality.

Without prior knowledge of the dataset, predicting the optimal embedding dimension is non-trivial. A systematic approach involves testing two- or three-dimensional embeddings, evaluating cluster separation and structural preservation, and then considering higher dimensions if needed. In our study, three embedding dimensions provided well-separated and meaningful clusters, justifying our choice. Chemical bond analysis and clustering results demonstrate that this dimensionality reduction approach is optimal for our dataset. Choosing an embedding dimension higher than necessary preserves the original manifold’s characteristics but does not improve interpretability while increasing computational cost. Conversely, reducing dimensions below the optimal level results in significant information loss and poorly separated clusters.

### Selection of descriptors

We selected 15 descriptors based on their chemical relevance for sulfur-nitrogen bonding and their general utility for spotting unusual interactions. However, our framework can accommodate any number of descriptors, so new features can be added as needed. By design, the algorithm imposes no strict limit on how many descriptors it can handle, allowing us to integrate domain-specific knowledge or adapt to new experimental methods.

Overall, focusing on a small, carefully chosen descriptor set and embedding it in at least three dimensions helps us balance clarity, speed, and analytical depth, making our approach both scalable and well-suited for uncovering subtle covalent modifications in large protein structure databases.

### Quantum-mechanical calculations

To further corroborate the finding of new NOS linkages suggested by our *SimplifiedBondfinder* algorithm, we employ quantum-mechanical calculations as a guide to acquire structural insight during manual inspection of suggested probable candidates for NOS linkages.

To be more precise, we have performed geometry optimization for prospective candidates of NOS linkages in Lys-NOS-Cys, Gly-NOS-Cys, ARG-N_*η*_OS-Cys, and ARG-N_*ε*_OS-Cys complexes. The software package Gaussian16 - A.03 (Gaussian 16, revision C.01)^44^ is used for geometry optimization in water with the B3LYP-D3(BJ)/def2-TZVPD level of theory^45–47^. The following geometrical parameters were calculated for the optimized structures: the distance between the sulfur and nitrogen atoms (dist(*S*, *N*)), and the angles (${\measuredangle}_{{\rm{CSN}}}$, ∡_CNS_, ∡_NOS_).

### Preparation of residue termini and geometry optimization

To accurately model the electronic structure of the selected residues within a truncated peptide fragment, we manually capped the termini in a chemically neutral form to eliminate artificial charge effects. Specifically, at the N-terminal truncation site, the backbone nitrogen was saturated with a hydrogen atom, resulting in a neutral amine (-NH_2_) group. At the C-terminal truncation site, the backbone carbon was modified to retain a double bond to oxygen (C=O) while being saturated with a hydrogen atom, yielding a formyl (-COH) moiety. This approach ensures a well-defined electronic environment suitable for quantum mechanical calculations while avoiding the introduction of artificial charges that could distort the system’s electronic structure.

In the case of 6PGD, the cysteine residue is naturally terminal in the peptide sequence. At the N-terminal position, the backbone nitrogen was assigned a neutral amine (-NH_2_) configuration, consistent with the capping approach used in other systems. At the C-terminal position, the carboxyl group (-COO^−^) was protonated at one of the oxygen atoms, yielding the neutral carboxyl (-COOH) form. This ensures that the termini maintains an overall neutral charge, providing a chemically consistent environment for quantum mechanical calculations.

To maintain the backbone conformation of the residue within a realistic structural environment, we applied positional restraints during the quantum mechanical geometry optimization. Specifically, for all systems, all backbone atoms (C, N, O) were kept fixed to preserve the original peptide geometry. Additionally, in the case of the cysteine residue in 6PGD, the terminal oxygen atom was also constrained to maintain the integrity of the C-terminal carboxyl (-COOH) group. These constraints were applied to prevent artifacts arising from excessive relaxation in an isolated fragment, ensuring that the optimized geometries remain representative of their native conformations in the full protein structure.

The Gly-NOS-Cys linkage was optimized with the nitrogen atom of the NOS bridge modeled in two distinct protonation states, revealing that these variations exert minimal influence on key structural parameters. Similarly, the ARG-N_*η*_OS-Cys configuration, with N_*η*1_ participating in the NOS linkage, was optimized across three protonation states, while the ARG-N_*ε*_OS-Cys variant was assessed in three. Structural metrics, including dist(S, N), ${\measuredangle}_{{\rm{CSN}}}$, ∡_CNS_, and ∡_NOS_, were systematically evaluated across these configurations (see Table 1 for details). For non-NOS complexes, protonation states are detailed in Supplementary Table VI. All protonation states were included in our calculations to ensure comprehensive analysis, irrespective of their biological relevance.

### NOS Bond modeling and refinement

To validate the presence of NOS covalent linkages predicted by our clustering approach, we re-refined four representative protein structures using phenix.refine (version 1.20.1-4487-000). For each structure, an initial pre-refinement phase (10 cycles until convergence of *R*_work_ and *R*_free_ over three successive rounds) was followed by five cycles of standard refinement incorporating XYZ coordinate refinement, TLS parameterization, occupancy refinement, and individual B-factor optimization. Tailored geometric restraints for the NOS bond were generated from quantum mechanical calculations, and partial occupancies were refined where applicable.

Comprehensive structure validation was performed using phenix.molprobity to assess geometric quality, clash scores, and steric interactions, ensuring consistency with high-resolution crystallographic data. Additionally, phenix.table1 was used to generate a complete validation report, summarizing refinement statistics, model quality metrics, and stereochemical deviations. These validation steps confirmed the structural integrity of the NOS linkages and their compatibility with electron density maps. Full details of the refinement procedures, validation statistics, and geometric parameters for all four structures are provided in the Supporting Information.

AI-empowered tools have been employed to improve the language and readability of parts of this manuscript. All scientific content was generated, reviewed, and approved by the authors.

## Supplementary information

Supplementary Material

Description of Additional Supplementary Files

Supplementary Data 1

Supplementary Code 1

Supplementary Code 2

nr-reporting-summary.pdf

## Data Availability

The data supporting the findings of this study are available within the paper and its Supplementary Information files. Raw data are available via GitHub at SimplifiedBonfinder GitHub repository. The structures cited in this publication are available under their respective PDB accession codes. Raw protein refinement data and validation statistics are available at Protein Refinement Data. Raw data corresponding to Quantum-mechanical calculations and RMSD calculations are available at Quantum-mechanical Data.

## Code availability

The SimplifiedBondfinder algorithm and python scripts used for generating figures are available on SimplifiedBonfinder GitHub repository.
