# Supplementary material for: Revealing arginine-cysteine and glycine-cysteine NOS linkages by a systematic re-evaluation of protein structures
Source: Commun Chem. 2025 May 13;8:146. doi: 10.1038/s42004-025-01535-w (PMC12075730; doi:10.1038/s42004-025-01535-w)
Supplement: Supplementary file 1 — Supplementary Material [file 42004_2025_1535_MOESM1_ESM.pdf]

Supplementary information for
Revealing arginine–cysteine and glycine–cysteine NOS linkages by a systematic re-evaluation of protein structures

Sophia Bazzi*[1] and Sharareh Sayyad[2,3]

[1] *Institute of Physical Chemistry, Georg-August University Göttingen, Tammannstraße 6, Göttingen, D-37077, Germany**

[2] *Department of Mathematics and Statistics, Washington State University, Pullman, Washington 99164-3113, USA*

[3] *Mathematical Institute, Georg-August University Göttingen, Bunsenstraße 3-5, Göttingen, 37073, Germany*

Contents

Supplementary Figures 1
- Pair plots and UMAP results obtained from the SimplifiedBondfinder algorithm 1
- Pair plot for Lys-NOS-Cys linkages 4
- Pair plot for Gly–NOS–Cys linkages 5
- Density distribution of reference Cys-NOS-Lys linkages 6

Supplementary Tables 7
- Frequency of N-S contacts by amino acid pairs in PDB-REDO 7
- Bonding environments within the Cys-Gly pairs 9
- Structural parameters of NOS-linked lysine and arginine complexes 10
- Newly found NOS linkages between cysteine sulfur and lysine nitrogen in 65 pdb structures 10
- Structural parameters and RMSD for Gly-Cys and Arg-Cys complexes without an NOS linkage 11
- Gibbs free energy change (ΔG) for NOS-linked arginine and glycine complexes 11
- X-ray crystallographic structural parameters of novel NOS linkages 12

Refinement and Validation of NOS Linkages 13

References 15

Supplementary Figures

Pair plots and UMAP results obtained from the SimplifiedBondfinder algorithm

Figure S1 (A)(a)-(f) illustrates the pair plot for Lys-NOS-Cys linkages. The neighboring residues are collected as sets of amino acid residues (see Extended data). In our algorithm, we numerize these sets by performing hashing on each set, ensuring that the permutation of elements in the set results in the same final hashed number. Clearly, the exact value of these hashed numbers is not of significance. The x-axes in Figs. S1 (A)(c) and (A)(f) represent the sets of neighboring residues as the map from the sets to hashed numbers is one-to-one. We do not observe a distinct pattern in neighboring residues that can discriminate between the probable and improbable samples. This does not necessarily imply that neighboring residues do not play a role in facilitating NOS formation. Rather, based on the criteria selected, no discriminating difference between the neighboring residues in probable and improbable samples was identified. Additionally, when examining the $\mathrm{Ngb}^{\mathrm{S}}$ and $\mathrm{Ngb}^{\mathrm{N}}$ among the reference points (see Figure S1 in Supplementary information), no common pattern emerged that suggests specific neighboring residues are associated with the experimentally verified samples.

Unlike the Lys-NOS-Cys samples, the majority of samples of the probable and improbable clusters in Figure S1 (B)(a) possess similar values of $\mathrm{Bfac}^{\mathrm{S}}_{\mathrm{BDB}}$. Similar to $\mathrm{Bfac}^{\mathrm{S}}_{\mathrm{BDB}}$, for $\mathrm{dist}(S, N)$ in Figure S1 (B)(c), the modes of distributions for probable and improbable clusters are very close. Additionally, we hardly can

deduce details from the correlation between these $\text{Bfac}^{\text{S}}_{\text{BDB}}$ and $\text{dist}(S, N)$ in (B)(b), except the dominance of probable samples at $\text{dist}(S, N) < 2.75\mathring{A}$. This brings us to the conclusion that these two parameters are individually insufficient to distinguish probable from improbable clusters.

The pair plot of the four-dimensional (descriptor) space in the case of ARG-N$_\eta$OS-CYS is presented in Supplementary Figure S1 (C)(a)-(j). Panel (C)(a) shows the density plot for the values of $\text{SASA}^{\text{N}}_{\text{atom}}$. The mode of distribution for all three clusters centers at around 0.0 $\mathring{A}^2$ to 1.25 $\mathring{A}^2$. It is clear that the mode of the improbable cluster is similar to the mode of the probable clusters, resulting in the fact that values of $\text{SASA}^{\text{N}}_{\text{atom}}$ alone cannot sufficiently discriminate cases with a high probability of hosting Arg-N$_\eta$OS-Cys linkages.

In the case of Arg-N$_\varepsilon$OS-Cys, the algorithm selects a set of five descriptors. When angles $\angle_{\text{CSN}}$ (D)(g)-(D)(j) and $\angle_{\text{CNS}}$ (D)(k)-(D)(o) are taken into account, we witness a clear mode separation in the distributions of clusters. However, despite the difference in modes of the two clusters, the complete overlap in the overall distributions of $\angle_{\text{CNS}}$ (D)(k)-(D)(o) indicates that this angle alone cannot predict the likelihood of forming Arg-N$_\varepsilon$OS-Cys linkages. The calculated values of $\angle_{\text{CNS}}$ from Table IV are 126.81°, 123.21°, and 122.62°. The correlations between angles, and particularly $\angle_{\text{CSN}}$, with ($\text{Bfac}^{\text{S}}_{\text{BDB}}$, SASA^{S}, $\text{SASA}^{\text{N}}_{\text{atom}}$) in panels (g)-(n) provide the possibility of separating clusters of probable and improbable candidates from each other.

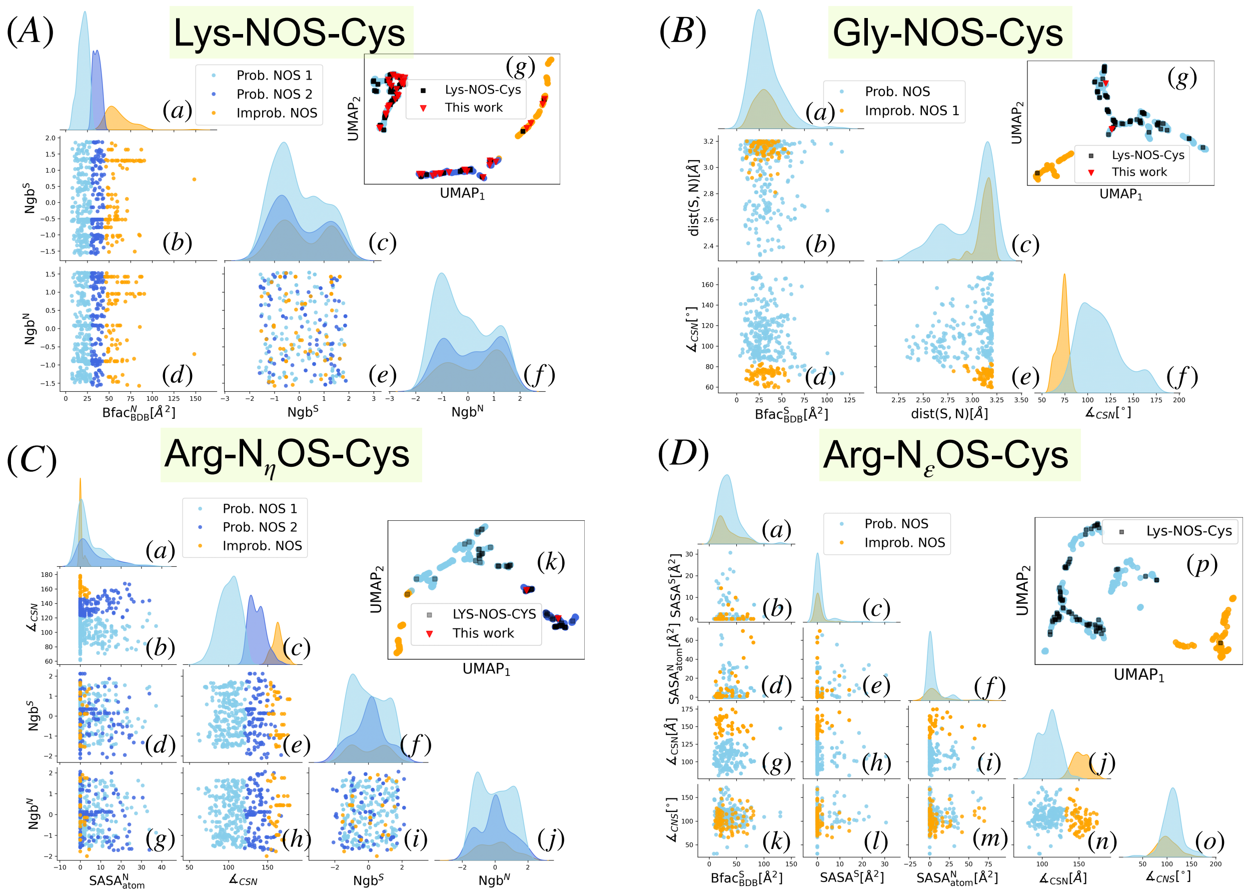

Supplementary Figure S1: **Pair plots and UMAP results obtained from the SimplifiedBondfinder algorithm.** Pair plots and UMAP plots for covalent linkages of Lys-NOS-Cys (A), Gly-NOS-Cys (B), Arg-N$_\eta$OS-Cys (C), and Arg-N$_\varepsilon$OS-Cys (D). Probable NOS-bond candidates are depicted in sky blue and royal blue, while improbable candidates are shown in orange. The black square points denote the reference dataset presented in Ref. [1]. The red inverted triangles depict our newly found sets of proteins exhibiting NOS linkages. For the Lys-NOS-Cys dataset, each cluster in (A)(g) consists of $n = 271$ (sky blue), $n = 155$ (royal blue), and $n = 101$ (orange) samples. The Silhouette score in (A)(g) is 0.61. The mode of $\mathrm{Bfac}^{\mathrm{N}}_{\mathrm{BDB}}$ for probable samples in 22.21 (sky blue) and 36.24 (royal blue) and 52.84 (orange) in panel (A)(a). The most frequent set of neighbors to the sulfur atom for hashed values in (A)(c) and for each cluster is $\{\mathrm{GLY}, \mathrm{THR}\}$ (sky blue and royal blue) and $\{\mathrm{ALA}, \mathrm{GLU}, \mathrm{ASP}\}$ (orange). Most common sets of nitrogen atom neighbors after unnumerizing values in (A)(f) read $\{\mathrm{ARG}, \mathrm{SER}\}$ (sky blue and royal blue) and $\{\mathrm{GLY}, \mathrm{ILE}, \mathrm{VAL}\}$ (orange). For Gly-NOS-Cys in (B), the cluster sizes are $n = 271$ (sky blue), and $n = 81$ (orange). The Silhouette score is 0.58. Dominant modes of density distributions for $\{\mathrm{Bfac}^{\mathrm{S}}_{\mathrm{BDB}}, \mathrm{dist}(S, N), \angle_{\mathrm{CSN}}\}$ for (sky blue, orange) samples are $\{(24.56, 29.91), (3.16, 3.17), (96.63, 74.68)\}$ in panels (B)(a), (B)(c) and (B)(f), respectively. For Arg-N$_\eta$OS-Cys in (C), the sizes of generated clusters are $n = 266$ (sky blue), $n = 147$ (royal blue), and $n = 54$ (orange). The associate Silhouette score is 0.55. The dominant modes of density distributions for $\mathrm{SASA}^{\mathrm{N}}_{\mathrm{atom}}$ (C)(a) and $\angle_{\mathrm{CSN}}$ (C)(c) for (sky blue, royal blue, orange) samples are (0.46, 1.24, 0.00) and (106.79, 128.61, 162.92), respectively. The most frequent set of neighbors to the sulfur atom for each cluster, after unnumerizing the values, in (C)(f) for each cluster reads $\{\mathrm{ILE}, \mathrm{LEU}\}$ (sky blue), $\{\mathrm{GLN}, \mathrm{HIS}, \mathrm{TRP}\}$ (royal blue) and $\{\mathrm{THR}, \mathrm{GLN}, \mathrm{LEU}\}$ (orange). The most common set of neighbors to the nitrogen atom, after unhashing values, for each cluster in (C)(j) are $\{\mathrm{ARG}\}$ (sky blue), $\{\mathrm{GLY}, \mathrm{ARG}, \mathrm{VAL}, \mathrm{LEU}, \mathrm{TRP}\}$ (royal blue) and $\{\mathrm{ARG}, \mathrm{GLY}, \mathrm{PRO}, \mathrm{SER}\}$ (orange). For Arg-N$_\varepsilon$OS-Cys dataset in (D), the size of clusters read $n = 235$ (sky blue) and $n = 83$ (orange). The associate Silhouette score is 0.56. The dominant modes of density distributions in $\mathrm{Bfac}^{\mathrm{S}}_{\mathrm{BDB}}$ (D)(a), $\mathrm{SASA}^{\mathrm{S}}$ (D)(c), $\mathrm{SASA}^{\mathrm{N}}_{\mathrm{atom}}$ (D)(f), $\angle_{\mathrm{CSN}}$ (D)(j) and $\angle_{\mathrm{CNS}}$(D)(o) for (probable, improbable) samples are (0.08, 0.12), (0.56, 2.40), (32.47, 20.53), (113.38,148.07), and (111.81, 96.63), respectively.

Pair plot for Lys-NOS-Cys linkages

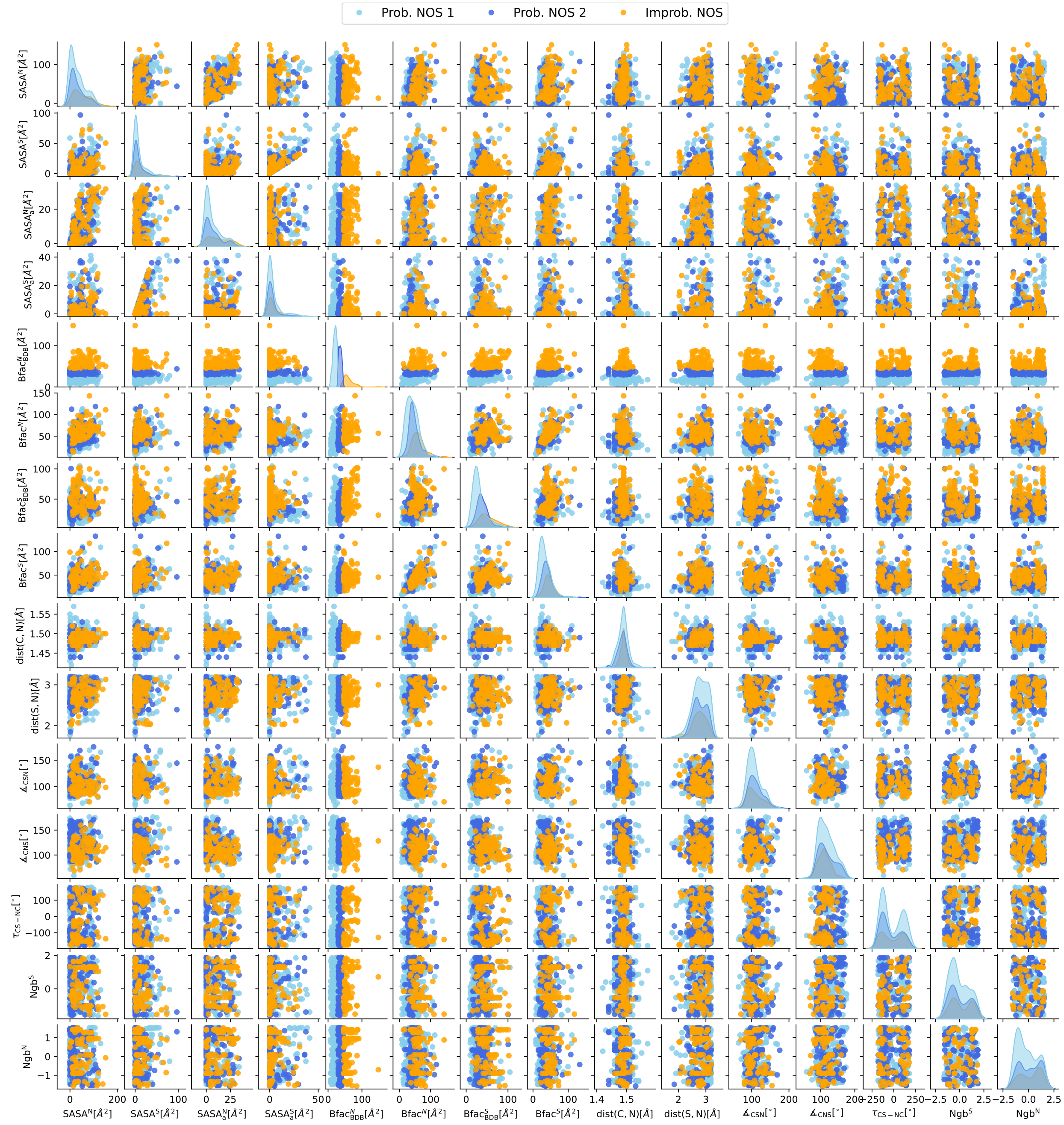

Supplementary Figure S2: **Pair plot for Lys-NOS-Cys linkages.** Probable NOS-bond candidates appear in sky blue and royal blue, while improbable candidates are in orange. The 15 descriptors arise from the minimal descriptor set of the SimplifiedBondfinder algorithm, including the nitrogen atom's B-factor ($\mathrm{Bfac}^{\mathrm{N}}_{\mathrm{BDB}}$) and the neighboring residues within a 4 Å radius of the C_{α} atoms of lysine ($\mathrm{Ngb}^{\mathrm{N}}$) and cysteine ($\mathrm{Ngb}^{\mathrm{S}}$).

Pair plot for Gly–NOS–Cys linkages

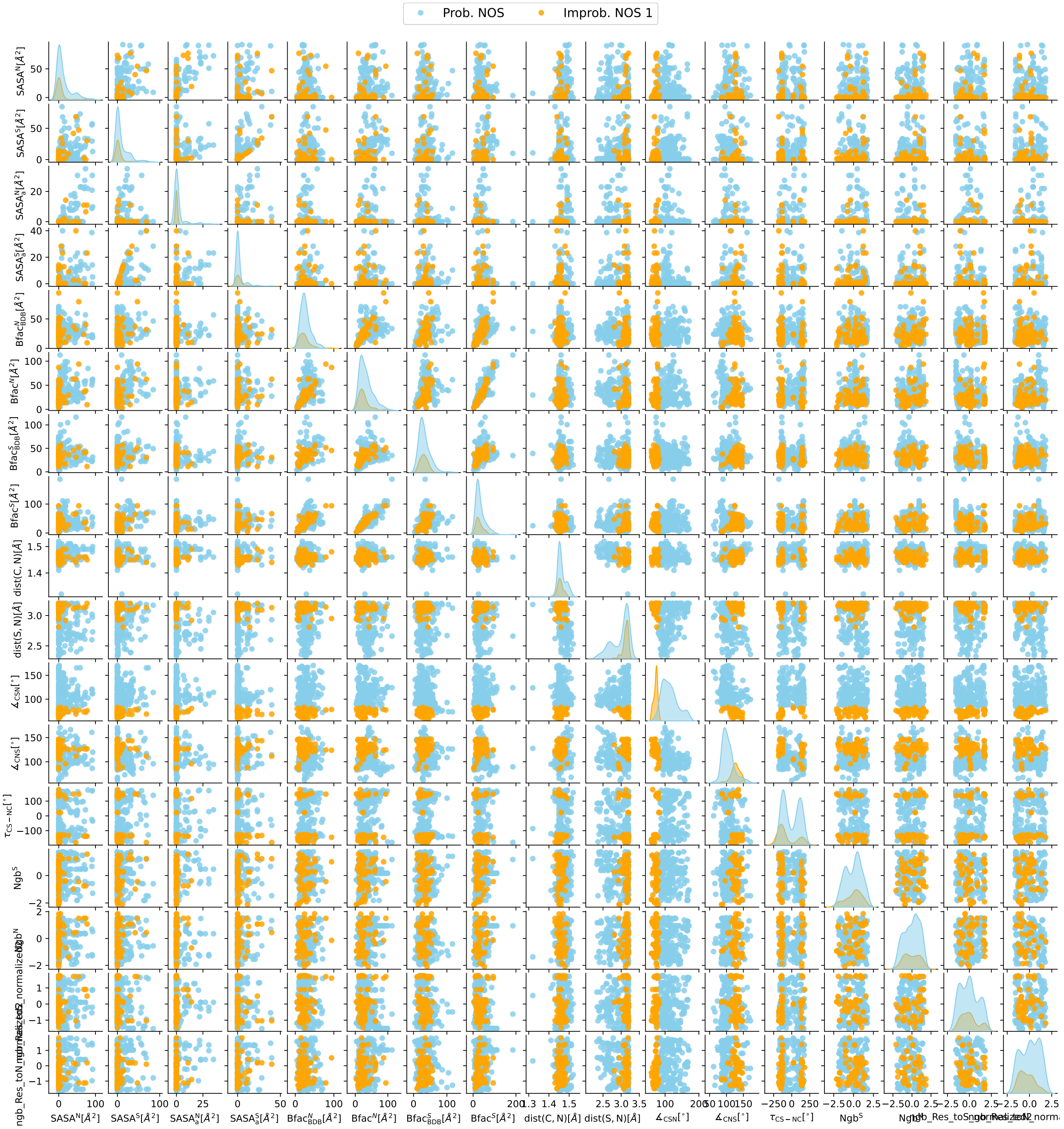

Supplementary Figure S3: **Pair plot for Gly–NOS–Cys linkages.** Probable NOS-bond candidates appear in sky blue, while improbable candidates are shown in orange. The descriptor set includes the B-factor for the sulfur-bearing residue ($\mathrm{Bfac}^{\mathrm{S}}_{\mathrm{BDB}}$), the sulfur-nitrogen distance $\mathrm{dist}(S, N)$, and the angle $\angle_{\mathrm{CSN}}$.

Density distribution of reference Cys-NOS-Lys linkages

In our approach, we took advantage of the previously identified Lys-NOS-Cys linkages as reference points. To better understand the distribution of these reference samples for different descriptors, we present the Supplementary Figure S4. The distributions exhibit peaks at 3.56 ($\mathrm{SASA}^{\mathrm{N}}[\AA^2]$), 1.08 ($\mathrm{SASA}^{\mathrm{S}}[\AA^2]$), 0.39 ($\mathrm{SASA}^{\mathrm{N}}_{\mathrm{atom}}[\AA^2]$), 0.41 ($\mathrm{SASA}^{\mathrm{S}}_{\mathrm{atom}}[\AA^2]$), 24.30 ($\mathrm{Bfac}^{\mathrm{N}}_{\mathrm{BDB}}$), 36.88 ($\mathrm{Bfac}^{\mathrm{N}}_{\mathrm{BDB}}$), 30.32 ($\mathrm{Bfac}^{\mathrm{S}}_{\mathrm{BDB}}$), 23.48 ($\mathrm{Bfac}^{\mathrm{S}}_{\mathrm{BDB}}$), 1.49 ($\mathrm{dist}(C,N)[\AA]$), 2.67 ($\mathrm{dist}(S,N)[\AA]$), 105.22 ($\angle_{\mathrm{CSN}}[°]$), 101.32 ($\angle[°]$), -119.48 (τ_{CS-NC}).

We also note that the neighboring residues set of $\{\mathrm{TRP}, \mathrm{GLY}, \mathrm{SER}\}$ for the sulfur atom appears four times for different samples in the reference dataset. For the nitrogen atom, four sets of neighboring residues, with four times occurrence of each set, are $\{\mathrm{TYR}, \mathrm{GLU}, \mathrm{ASN}, \mathrm{VAL}\}$, $\{\mathrm{ALA}, \mathrm{PRO}\}$, $\{\mathrm{ILE}, \mathrm{LEU}, \mathrm{VAL}\}$, $\{\mathrm{ILE}, \mathrm{GLY}, \mathrm{VAL}\}$.

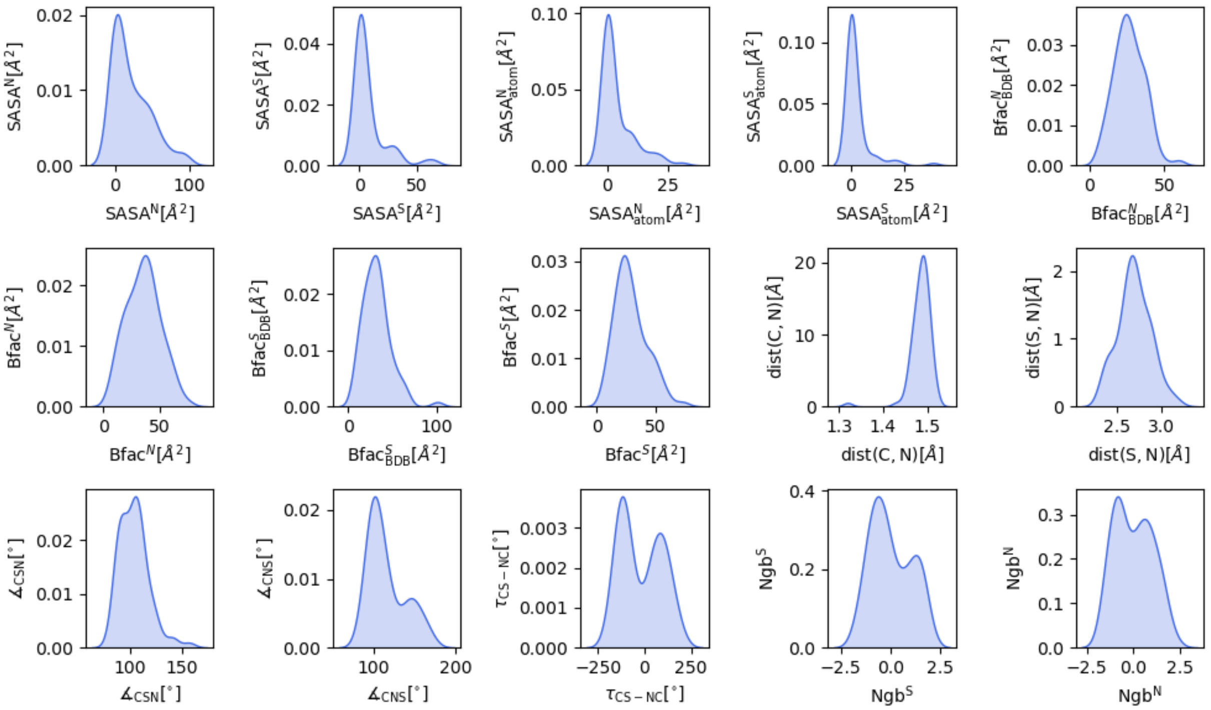

Supplementary Figure S4: **Density distributions of structural parameters in experimentally reported NOS linkages.** The reference NOS linkages reflect data from Ref. [1], with modes at 3.56 ($\mathrm{SASA}^{\mathrm{N}}[\AA^2]$), 1.08 ($\mathrm{SASA}^{\mathrm{S}}[\AA^2]$), 0.39 ($\mathrm{SASA}^{\mathrm{N}}_{\mathrm{atom}}[\AA^2]$), 0.41 ($\mathrm{SASA}^{\mathrm{S}}_{\mathrm{atom}}[\AA^2]$), 24.30 ($\mathrm{Bfac}^{\mathrm{N}}_{\mathrm{BDB}}$), 36.88 ($\mathrm{Bfac}^{\mathrm{N}}$), 30.32 ($\mathrm{Bfac}^{\mathrm{S}}_{\mathrm{BDB}}$), 23.48 ($\mathrm{Bfac}^{\mathrm{S}}$), 1.49 ($\mathrm{dist}(C,N)[\AA]$), 2.67 ($\mathrm{dist}(S,N)[\AA]$), 105.22 ($\angle_{\mathrm{CSN}}[°]$), 101.32 ($\angle[°]$), -119.48 (ϕ_{CS-NC}). The most frequent neighboring residues for sulfur are $\{\mathrm{CYS}, \mathrm{TRP}, \mathrm{GLY}, \mathrm{SER}\}$ while nitrogen exhibits four common sets: $\{\mathrm{LYS}, \mathrm{TYR}, \mathrm{GLU}, \mathrm{ASN}, \mathrm{VAL}\}$, $\{\mathrm{LYS}, \mathrm{ALA}, \mathrm{PRO}\}$, $\{\mathrm{ILE}, \mathrm{LYS}, \mathrm{LEU}, \mathrm{VAL}\}$, $\{\mathrm{ILE}, \mathrm{LYS}, \mathrm{GLY}, \mathrm{VAL}\}$.

Supplementary Tables

This section compiles the comprehensive results underpinning the main text, organized across the supplementary tables. These tables include:

(i) Frequency distributions of S–Amide N and S–Side-chain N contacts in the PDB-REDO dataset (Supplementary Tables I–II).

(ii) Bonding environments within the "improbable" cluster (Supplementary Table III).

(iii) Quantum mechanically optimized structural parameters for NOS-linked complexes (Supplementary Table IV).

(iv) PDB filenames for 65 identified Lys–NOS–Cys linkages (Supplementary Table V).

(v) Structural parameters and root-mean-square deviations (RMSDs) for non-NOS complexes (Supplementary Table VI).

(vi) Gibbs free energy changes for NOS formation (Supplementary Table VII).

(vii) Four additional tables summarizing refinement improvements in selected cases (Supplementary Tables IX–XII).

(viii) X-ray crystallographic parameters for proposed NOS linkages (Supplementary Table VIII).

Frequency of N-S contacts by amino acid pairs in PDB-REDO

Supplementary Tables I and II provide a comprehensive analysis of 23,129 sulfur-nitrogen (S-N) contacts identified in the PDB-REDO dataset. These contacts are systematically categorized by the sulfur-containing residue (cysteine or methionine) and its partner residue, distinguishing between interactions with backbone nitrogen (Amide N) and side-chain nitrogen (Side-chain N). For this study, only residue pairs with available B-factor data in the Biomolecular Database (BDB) were selected for further analysis, ensuring consistent B-factor values across all residue types. Our investigation focuses primarily on the sulfur atom of cysteine, given its critical role in potential NOS linkage formation.

S-Amide N contacts:

In the case of cysteine sulfur and Amide N interactions, CYS-CYS contacts are the most frequent (2,598 cases). However, considering that their proximity could be due to disulfide bridges, we have excluded them from further investigation in the current study. It is worth noting that some of these interactions may indicate potential non-traditional NOS bonding, which will be explored in future investigations. For the present study, we focus on the next most frequent S-Amide N contact: CYS-GLY (1,138 cases).

S-Side-chain N contacts:

For interactions between the sulfur atom of cysteine and side chain nitrogen atoms, cysteine-arginine pairs are the most common (1,608 cases), followed by cysteine-histidine (1,416 cases) and cysteine-lysine pairs (1,214 cases). We have chosen to exclude cysteine-histidine contacts from further investigations in this study because histidine is frequently involved in metal coordination [2, 3]. The cysteine-arginine and cysteine-lysine contacts will be the primary focus of our current analysis, while the remaining contacts will be investigated in future studies. Methionine interactions, though not the focus of this study, show distinct patterns from cysteine and may offer additional insights in future investigations.

Considering these focused selections enables us to prioritize our investigation on the most promising candidates for novel NOS linkages while acknowledging the potential for more comprehensive research frameworks in succeeding studies.

Supplementary Table I: Distribution of S-Amide N contacts

Cysteine			Methionine		
S-residue	Partner	Frequency	S-residue	Partner	Frequency
Cysteine	CYS	2598	Methionine	TYR	474
	GLY	1138		GLY	468
	GLN	861		ALA	305
	HIS	857		LEU	230
	ALA	643		PHE	150
	SER	550		VAL	142
	THR	433		ILE	137
	ASP	432		LYS	123
	GLU	415		SER	115
	LYS	367		THR	101
	ARG	366		GLU	95
	TRP	364		ASP	94
	VAL	344		ARG	85
	MET	321		PRO	78
	LEU	316		ASN	70
	ASN	274		GLN	62
	PHE	272		HIS	50
	TYR	248		MET	49
	ILE	174		TRP	38
	PRO	97		CYS	20

Supplementary Table II: **Distribution of S-Side-chain N contacts.** For Cysteine–ARG contacts (1608 total), 996 involve N^{η} (NH1 or NH2) and 612 involve N^{ε} (NE). For Methionine–ARG contacts (1498 total), 1098 involve N^{η} and 400 involve N^{ε}.

S-residue	Partner	Frequency	S-residue	Partner	Frequency
Cysteine	ARG	1608<br>996 (N_{η})<br>612 (N_{ε})	Methionine	ARG	1498<br>1098 (N_{η})<br>400 (N_{ε})
	HIS	1416		HIS	668
	LYS	1214		GLN	517
	TRP	1174		TRP	332
	GLN	490		LYS	256

Bonding environments within the Cys-Gly pairs

Supplementary Table III: **Bonding environments within the Cys-Gly pairs.** The sulfur atom of cysteine in this cluster frequently engages in diverse interactions, including metal coordination, disulfide bonds, and five-membered ring structures. These covalent environments likely contribute to the intermediate values of $\mathrm{Bfac}^{\mathrm{S}}_{\mathrm{BDB}}$.

**Cys-Gly Pair**	**Type of Interaction**
4a2c_A_CYS_SG_38_A_GLY_N_39.pdb	metal
4a2c_B_CYS_SG_38_B_GLY_N_39.pdb	metal
7aas_A_CYS_SG_178_A_GLY_N_179.pdb	metal
7aas_B_CYS_SG_178_B_GLY_N_179.pdb	metal
7aas_D_CYS_SG_178_D_GLY_N_179.pdb	metal
7aas_E_CYS_SG_178_E_GLY_N_179.pdb	metal
5ac3_A_CYS_SG_200_A_GLY_N_201.pdb	disulfide
3atq_A_CYS_SG_47_A_GLY_N_48.pdb	5-membered ring-structure
4ay7_A_CYS_SG_319_A_GLY_N_320.pdb	metal
4ay7_B_CYS_SG_319_B_GLY_N_320.pdb	metal
7b28_C_CYS_SG_144_C_GLY_N_145.pdb	disulfide
2bkr_A_CYS_SG_163_A_GLY_N_164.pdb	C-S bond
6c49_A_CYS_SG_153_A_GLY_N_154.pdb	metal
4d5g_A_CYS_SG_428_A_GLY_N_429.pdb	5-membered ring-structure
4d5g_B_CYS_SG_428_B_GLY_N_429.pdb	5-membered ring-structure
3efy_B_CYS_SG_109_B_GLY_N_110.pdb	5-membered ring-structure
6eib_A_CYS_SG_72_A_GLY_N_73.pdb	di-sulfide
6eib_B_CYS_SG_72_B_GLY_N_73.pdb	di-sulfide
6eib_C_CYS_SG_72_C_GLY_N_73.pdb	di-sulfide
6eib_D_CYS_SG_72_D_GLY_N_73.pdb	di-sulfide
6esk_A_CYS_SG_106_A_GLY_N_107.pdb	5-membered ring-structure
6esv_A_CYS_SG_106_A_GLY_N_107.pdb	5-membered ring-structure
7f27_B_CYS_SG_339_B_GLY_N_340.pdb	5-membered ring-structure
3gqj_A_CYS_SG_123_A_GLY_N_124.pdb	5-membered ring-structure
6h0c_A_CYS_SG_367_A_GLY_N_368.pdb	5-membered ring-structure
6h0d_A_CYS_SG_367_A_GLY_N_368.pdb	5-membered ring-structure
6h98_A_CYS_SG_370_A_GLY_N_371.pdb	5-membered ring-structure
6h99_A_CYS_SG_370_A_GLY_N_371.pdb	5-membered ring-structure
4ium_A_CYS_SG_270_A_GLY_N_271.pdb	C-S bond
4jbl_B_CYS_SG_309_B_GLY_N_310.pdb	5-membered ring-structure
4jji_A_CYS_SG_177_A_GLY_N_178.pdb	metal
6jow_A_CYS_SG_95_A_GLY_N_96.pdb	5-membered ring-structure
6jow_B_CYS_SG_95_B_GLY_N_96.pdb	5-membered ring-structure
6jow_C_CYS_SG_95_C_GLY_N_96.pdb	5-membered ring-structure
6jow_D_CYS_SG_95_D_GLY_N_96.pdb	5-membered ring-structure
5k6k_B_CYS_SG_291_B_GLY_N_292.pdb	di-sulfide, 5-membered ring
6ktw_A_CYS_SG_370_A_GLY_N_371.pdb	5-membered ring-structure
6ktw_A_CYS_SG_412_A_GLY_N_413.pdb	5-membered ring-structure
6ktz_A_CYS_SG_370_A_GLY_N_371.pdb	5-membered ring-structure
6kvy_A_CYS_SG_370_A_GLY_N_371.pdb	5-membered ring-structure
4l6h_A_CYS_SG_739_A_GLY_N_740.pdb	metal
4l6o_A_CYS_SG_739_A_GLY_N_740.pdb	metal

Structural parameters of NOS-linked lysine and arginine complexes

Supplementary Table IV: **Structural parameters of NOS-linked lysine and arginine complexes.** Selected structural parameters for the optimized complexes containing NOS linkage involving N_ε of arginine residue and nitrogen atom of lysine side chain.

Complex	dist(S,N) [Å]	$\angle_{CSN}$ [°]	$\angle_{CNS}$ [°]	$\angle_{NOS}$ [°]
Lys-NOS-Cys	2.62	96.33	122.40	113.45
Arg-N_εOS-Cys^{+1} ($N_\eta H_2$, $N_\eta H_2$)	2.60	95.96	126.81	113.76
ARG-N_εOS-Cys ($N_\eta H_2$, $N_\eta H$)	2.60	99.72	123.21	113.09
ARG-N_εOS-Cys^{+1} ($N_\eta H_3$, $N_\eta H$)	2.60	99.47	122.62	113.03

Newly found NOS linkages between cysteine sulfur and lysine nitrogen in 65 pdb structures

Supplementary Table V: **Newly found NOS linkages between cysteine sulfur and lysine nitrogen in 65 PDB structures.** Each entry specifies PDB ID, chain, residue type, atom name, residue number, and interaction partner details for cysteine sulfur (SG) and lysine nitrogen (NZ) contacts.

**Entry**	**Entry**	**Entry**
5ant_A_CYS_SG_87_A_LYS_NZ_130.pdb	5ant_B_CYS_SG_87_B_LYS_NZ_130.pdb	5ant_B_CYS_SG_104_C_LYS_NZ_114.pdb
8czw_B_CYS_SG_22_B_LYS_NZ_61.pdb	7ds0_A_CYS_SG_147_A_LYS_NZ_135.pdb	7ds1_A_CYS_SG_147_A_LYS_NZ_135.pdb
7e2q_A_CYS_SG_408_A_LYS_NZ_151.pdb	8e5z_B_CYS_SG_22_B_LYS_NZ_61.pdb	7e6k_A_CYS_SG_22_A_LYS_NZ_61.pdb
8e63_B_CYS_SG_22_B_LYS_NZ_61.pdb	8e68_B_CYS_SG_22_B_LYS_NZ_61.pdb	3etc_A_CYS_SG_298_A_LYS_NZ_256.pdb
3etc_B_CYS_SG_298_B_LYS_NZ_256.pdb	6fcd_A_CYS_SG_149_A_LYS_NZ_307.pdb	3fd6_B_CYS_SG_31_B_LYS_NZ_27.pdb
4fvr_A_CYS_SG_747_A_LYS_NZ_650.pdb	3ho2_A_CYS_SG_375_A_LYS_NZ_319.pdb	5i9u_A_CYS_SG_612_A_LYS_NZ_633.pdb
5iwg_B_CYS_SG_327_B_LYS_NZ_55.pdb	7k0h_B_CYS_SG_22_B_LYS_NZ_61.pdb	7l25_A_CYS_SG_69_A_LYS_NZ_133.pdb
7l25_B_CYS_SG_69_B_LYS_NZ_133.pdb	7lkr_B_CYS_SG_22_B_LYS_NZ_61.pdb	7lzu_B_CYS_SG_22_B_LYS_NZ_61.pdb
7lzx_B_CYS_SG_22_B_LYS_NZ_61.pdb	7m01_B_CYS_SG_22_B_LYS_NZ_61.pdb	7m02_B_CYS_SG_22_B_LYS_NZ_61.pdb
7m03_B_CYS_SG_22_B_LYS_NZ_61.pdb	4ndb_A_CYS_SG_24_A_LYS_NZ_41.pdb	4ndb_B_CYS_SG_24_B_LYS_NZ_41.pdb
1nvq_A_CYS_SG_168_A_LYS_NZ_166.pdb	2od5_A_CYS_SG_79_A_LYS_NZ_62.pdb	7opg_A_CYS_SG_151_A_LYS_NZ_174.pdb
3ov8_A_CYS_SG_62_A_LYS_NZ_69.pdb	2q9p_A_CYS_SG_68_A_LYS_NZ_74.pdb	3q98_A_CYS_SG_330_A_LYS_NZ_270.pdb
7rc1_A_CYS_SG_22_A_LYS_NZ_61.pdb	7s6x_A_CYS_SG_22_A_LYS_NZ_61.pdb	7s6x_A_CYS_SG_44_A_LYS_NZ_61.pdb
7s71_A_CYS_SG_22_A_LYS_NZ_61.pdb	7t43_B_CYS_SG_22_B_LYS_NZ_61.pdb	7t44_B_CYS_SG_22_B_LYS_NZ_61.pdb
7ubk_A_CYS_SG_108_B_LYS_NZ_63.pdb	7ubk_B_CYS_SG_108_A_LYS_NZ_63.pdb	1uku_A_CYS_SG_29_A_LYS_NZ_56.pdb
2uz8_B_CYS_SG_147_B_LYS_NZ_166.pdb	5v38_A_CYS_SG_1272_A_LYS_NZ_1228.pdb	5v38_B_CYS_SG_1272_B_LYS_NZ_1228.pdb
3vb5_A_CYS_SG_22_A_LYS_NZ_61.pdb	3vb5_B_CYS_SG_22_B_LYS_NZ_61.pdb	7vu6_B_CYS_SG_22_B_LYS_NZ_61.pdb
6wo9_A_CYS_SG_68_A_LYS_NZ_74.pdb	6wob_A_CYS_SG_68_A_LYS_NZ_74.pdb	6woc_A_CYS_SG_68_A_LYS_NZ_74.pdb
6wod_A_CYS_SG_68_A_LYS_NZ_74.pdb	6woe_A_CYS_SG_68_A_LYS_NZ_74.pdb	6woh_A_CYS_SG_68_A_LYS_NZ_74.pdb
5y4g_A_CYS_SG_223_A_LYS_NZ_275.pdb	5y4g_B_CYS_SG_223_B_LYS_NZ_275.pdb	5y7c_A_CYS_SG_223_A_LYS_NZ_275.pdb
5y7c_B_CYS_SG_223_B_LYS_NZ_275.pdb	5y84_A_CYS_SG_223_A_LYS_NZ_275.pdb	5y84_B_CYS_SG_223_B_LYS_NZ_275.pdb
4yhv_A_CYS_SG_445_A_LYS_NZ_443.pdb	3oy1_A_CYS_SG_283_A_LYS_NZ_346.pdb	

Structural parameters and RMSD for Gly-Cys and Arg-Cys complexes without an NOS linkage

Supplementary Table VI: **Structural parameters and RMSD for Gly-Cys and Arg-Cys complexes without an NOS linkage.** Selected structural parameters and root-mean-square deviations (RMSDs) for optimized Gly-Cys and Arg-Cys complexes without an NOS linkage. RMSD values were calculated between the optimized geometries and their corresponding PDB-REDO structures.

Complex	PDB ID	dist(S,N) [Å]	$\angle_{\mathrm{CSN}}$ [°]	$\angle_{\mathrm{CNS}}$ [°]	RMSD [Å]
Gly–Cys (NH_2, SH)	6PGD	4.26	110.17	75.79	0.5903
	6T3X	3.71	74.75	110.98	0.4640
Gly–Cys^{+1} (NH_3, SH)	6PGD	3.36	107.00	96.46	0.3135
	6T3X	3.40	78.47	111.84	0.3710
ARG–Cys ($N_\eta H$, $N_\eta H_2$, SH)	3MWB	3.64	151.98	99.35	0.9103
	3G2K	3.65	99.26	122.88	0.3167
ARG–Cys ($N_\eta H_2$, $N_\eta H$, SH)	3MWB	-	-	-	-
	3G2K	3.65	98.41	122.66	0.2726
ARG–Cys^{+1} ($N_\eta H_2$, $N_\eta H_2$, SH)	3MWB	3.68	143.05	93.42	0.8531
	3G2K	3.56	94.40	125.82	0.2858

In our optimization protocol, backbone atoms were fixed to their coordinates in the corresponding PDB-REDO structure except for glycine backbone nitrogen. This approach ensures that observed differences primarily reflect local side-chain rearrangements in RMSD calculations.

Gibbs free energy change (ΔG) for NOS-linked arginine and glycine complexes

Supplementary Table VII: **Gibbs free energy change (ΔG) for NOS-linked arginine and glycine complexes.** Values are reported for each PDB structure, with triplet O_2 calculations in parentheses.

Complex	PDB ID	ΔG [kcal/mol]
Gly-NOS-Cys (NH)	6PGD	-45.19 (-22.69)
	6T3X	-41.67 (-19.17)
Gly-NOS-Cys^{+1} (NH_2)	6PGD	-34.23 (-11.73)
	6T3X	-27.75 (-05.25)
ARG-N_ηOS-Cys ($N_{\eta 2}H_2$, $N_{\eta 1}$)	3MWB	-48.84 (-26.34)
	3G2K	-50.98 (-28.48)
ARG-N_ηOS-Cys^{+1} ($N_{\eta 2}H_2$, $N_{\eta 1}H_1$)	3MWB	-33.29 (-10.79)
	3G2K	-36.34 (-13.84)
ARG-N_ηOS-Cys ($N_{\eta 2}H$, $N_{\eta 1}H$)	3MWB	-43.38 (-20.88)
	3G2K	-43.40 (-20.90)

ΔG_{NOS} represents the Gibbs free energy change for the reactions $\mathrm{Arg + Cys + O_2 \rightarrow Arg(NOS)Cys + H_2O}$ and $\mathrm{Gly + Cys + O_2 \rightarrow Gly(NOS)Cys + H_2O}$. In NOS-linked products, an oxygen atom replaces a hydrogen on the amino group (glycine) or the $N_{\eta 1}$ site of the guanidinium group (arginine), forming the NOS linkage (see Fig. 1 in the main text).

X-ray crystallographic structural parameters of novel NOS linkages

Supplementary Table VIII presents X-ray crystallographic structural parameters, including bond lengths and angles, for the NOS linkages between glycine and cysteine and arginine and cysteine. These linkages were among those suggested by our algorithm as novel NOS linkages and were initially verified through manual inspection of their corresponding electron densities, as illustrated in Fig. 4 in the main paper. The structural parameters were obtained from the PDB-REDO [4] database and analyzed using the Coot software package [5]. Notably, these experimentally derived parameters agree with our quantum mechanical calculations of corresponding NOS linkages, as presented in Table 1 in the main text. This concordance between experimental and theoretical data further supports the validity of the proposed NOS linkages. A perfect agreement between these experimental values and our quantum-mechanical calculations is not expected, as NOS linkages have only recently been discovered.

Supplementary Table VIII: Structural parameters for new NOS linkages from PDB-REDO structures.

PDB code	Residue pair	dist(S,N) [Å]	∠CSN [°]	∠CNS [°]
6pgb	Cys334-Gly30, A	2.69	99.85	134.31
6T3X	Cys35-Gly-5, A	2.70	90.47	95.79
3MWB	Cys34-Arg43, B	2.89	101.33	109.83
3G2K	Cys783-Arg786, A	2.63	104.91	142.25

Refinement and Validation of NOS Linkages

To validate the presence of NOS covalent linkages predicted by our clustering approach, we re-refined four representative protein structures using `phenix.refine` (version 1.20.1-4487-000). For each structure, an initial pre–refinement phase (10 cycles until convergence of R_{work} and R_{free} over three successive rounds) was followed by 5 cycles of standard refinement incorporating XYZ coordinate refinement, TLS parameterization, occupancy refinement, and individual B-factor optimization. Tailored geometric restraints for the NOS bond were generated from quantum mechanical calculations, and partial occupancies were refined where applicable.

In all cases, the explicit modeling of the NOS linkage led to a marked improvement in the overall model fit, as evidenced by decreases in both R_{work} and R_{free} and reductions in the clashscore. In addition, inspection of the $2mF_o - DF_c$ electron density maps revealed that spurious positive difference density—previously observed in regions corresponding to the putative NOS bond—was largely eliminated after NOS modeling, leading to continuous electron density between the key residues.

Structure validation was performed using phenix.molprobity to assess geometric quality, clash scores, and steric interactions, ensuring consistency with high-resolution crystallographic data. Additionally, phenix.table1 was used to generate a complete validation report, summarizing refinement statistics, model quality metrics, and stereochemical deviations. Below, we provide a summary of the refinement and validation statistics for each protein, listed in the following order: 6PGD, 6T3X, 3MWB, and 3G2K. Detailed protocols and raw data are available at Protein Refinement Data.

6PGD: Refinement of Gly30–Cys334 NOS Linkage

For the 6PGD structure, pre-refinement revealed residual positive difference density between Gly30 and Cys334. Upon modeling the NOS bond, the unexplained density was removed and continuous electron density was observed connecting these residues. Table IX summarizes the key validation metrics.

Supplementary Table IX: **Refinement and validation statistics for 6PGD before and after NOS modeling.** Data represent the original model (PDB-REDO), before NOS (initial pre-refinement), and after NOS (final model).

Parameter	Original Model	Before NOS	After NOS	Change
Resolution range (Å)	33.67–1.50	33.67–1.50	33.67–1.50	No change
R_{work}	0.1516	0.1313	0.1312	Improved
R_{free}	0.1714	0.1534	0.1528	Improved
RMS(bonds) (Å)	0.017	0.005	0.005	Improved
RMS(angles) (°)	1.58	0.86	0.85	Improved
Ramachandran favored (%)	95.38	96.04	95.71	Slight decrease
Ramachandran outliers (%)	0.00	0.00	0.00	No change
Rotamer outliers (%)	0.00	0.00	0.00	No change
Clashscore	2.82	2.21	1.61	Improved
Average B-factor (Å^2)	12.40	12.58	12.85	Slight increase

6T3X: Refinement of Gly-5–Cys35 NOS Linkage

For the 6T3X structure, a prominent positive difference density was observed between the sulfur atom of Cys35 and the backbone nitrogen of Gly-5 in the pre-refinement state. Following NOS modeling, this residual density was eliminated, and the electron density map showed continuous connectivity between Gly-5 and Cys35. Table X details the improved refinement statistics.

Supplementary Table X: **Refinement and validation statistics for 6T3X before and after NOS modeling.** Data represent the original model (PDB-REDO), before NOS (initial pre-refinement), and after NOS (final model).

Parameter	Original Model	Before NOS	After NOS	Change
Resolution range (Å)	41.28–1.479	41.28–1.479	41.28–1.479	No change
R_{work}	0.1881	0.2218	0.2078	Improved
R_{free}	0.2254	0.2586	0.2516	Improved
RMS(bonds) (Å)	0.011	0.006	0.007	Improved
RMS(angles) (°)	1.28	0.79	0.92	Improved
Ramachandran favored (%)	99.23	98.97	98.97	Slight decrease
Ramachandran outliers (%)	0.00	0.00	0.00	No change
Rotamer outliers (%)	1.34	1.34	2.14	Increased
Clashscore	6.11	3.82	2.75	Improved
Average B-factor (Å^2)	38.86	37.94	38.49	Slight increase

3MWB: Refinement of Cys34–Arg43 NOS Linkage

In the 3MWB structure, a small but distinct positive difference density was observed between Cys34 and Arg43 prior to NOS modeling. After the introduction of the NOS bond, this residual density was eliminated, and the connectivity in the $2mF_o - DF_c$ map was markedly improved. Table XI summarizes the validation statistics for 3MWB.

Supplementary Table XI: **Refinement and validation statistics for 3MWB before and after NOS modeling.** Data represent the original model (PDB-REDO), before NOS (initial pre-refinement), and after NOS (final model).

Parameter	Original Model	Before NOS	After NOS	Change
Resolution range (Å)	42.28–1.999	42.28–1.999	42.28–1.999	No change
R_{work}	0.2020	0.1929	0.1852	Improved
R_{free}	0.2256	0.2337	0.2323	Improved
RMS(bonds) (Å)	0.010	0.007	0.008	Slight increase
RMS(angles) (°)	0.80	0.80	0.85	Slight increase
Ramachandran favored (%)	98.84	98.34	98.34	Minimal change
Rotamer outliers (%)	1.24	2.28	2.90	Increased
Clashscore	3.26	4.24	3.37	Improved
Average B-factor (Å^2)	35.12	32.68	32.82	Slight increase

3G2K: Refinement of Cys783–Arg786 NOS Linkage

In the 3G2K structure, the NOS bond between Cys783 and Arg786 was modeled following pre-refinement. Prior to NOS modeling, residual positive difference density was observed near the Arg786 side chain and between the interacting nitrogen and sulfur atoms. After introducing the NOS linkage, the electron density became continuous between Cys783 and Arg786 and the residual density was largely eliminated. The final model shows substantial improvements: R_{work} decreased from 0.1923 to 0.1436, R_{free} from 0.2122 to 0.1816, and the clashscore from 0.61 to 0.38. These improvements are summarized in Table XII.

Supplementary Table XII: **Refinement and validation statistics for 3G2K before and after NOS modeling.** Data represent the original model (PDB-REDO), before NOS (initial pre-refinement), and after NOS (final model).

Parameter	Original Model	Before NOS	After NOS	Change
Resolution range (Å)	25.75–2.001	25.25–2.001	25.75–2.001	Slight change
R_{work}	0.1923	0.1607	0.1436	Improved
R_{free}	0.2122	0.1930	0.1816	Improved
RMS(bonds) (Å)	0.011	0.007	0.008	Slight increase
RMS(angles) (°)	1.15	0.80	0.85	Slight increase
Ramachandran favored (%)	97.63	97.63	97.38	Slight decrease
Rotamer outliers (%)	1.00	1.14	0.86	Improved
Clashscore	0.61	0.83	0.38	Improved
Average B-factor (Å^2)	30.13	40.17	41.58	Increased

* *sophia.bazzi@uni-goettingen.de

[1] Fabian Rabe von Pappenheim, Marie Wensien, Jin Ye, Jon Uranga, Iker Irisarri, Jan de Vries, Lisa-Marie Funk, Ricardo A Mata, and Kai Tittmann, "Widespread occurrence of covalent lysine–cysteine redox switches in proteins," Nature Chemical Biology **18**, 368–375 (2022).

[2] K Ishara Silva, Brian C Michael, Steven J Geib, and Sunil Saxena, "ESEEM analysis of multi-histidine cu (ii)-coordination in model complexes, peptides, and amyloid-β," The Journal of Physical Chemistry B **118**, 8935–8944 (2014).

[3] Stefan Zechel, Martin D Hager, Tobias Priemel, and Matthew J Harrington, "Healing through histidine: Bioinspired pathways to self-healing polymers via imidazole–metal coordination," Biomimetics **4**, 20 (2019).

[4] Robbie P Joosten, Krista Joosten, Garib N Murshudov, and Anastassis Perrakis, "PDB_REDO: constructive validation, more than just looking for errors," Acta Crystallographica Section D: Biological Crystallography **68**, 484–496 (2012).

[5] Paul Emsley, Bernhard Lohkamp, William G Scott, and Kevin Cowtan, "Features and development of coot," Acta Crystallographica Section D: Biological Crystallography **66**, 486–501 (2010).
