# Supplementary material for: Revealing arginine-cysteine and glycine-cysteine NOS linkages by a systematic re-evaluation of protein structures
Source: Commun Chem. 2025 May 13;8:146. doi: 10.1038/s42004-025-01535-w (PMC12075730; doi:10.1038/s42004-025-01535-w)
Supplement: Supplementary file 2 — Description of Additional Supplementary Files [file 42004_2025_1535_MOESM2_ESM.pdf]

Description of Additional Supplementary Files

File name- Supplementary Data 1

File description- “Too-close contacts” from RCSB PDB validation reports for residue pairs investigated in this study.

File name- Supplementary Code 1

File description- “Python script for descriptor selection, dimensionality reduction, and clustering of candidate NOS linkages.”

File name- Supplementary Code 2

File description- “Python script for selection of high-confidence feature sets based on clustering performance.”
